# Functional characterization of *FABP3*, *5* and *7* gene variants identified in schizophrenia and autism spectrum disorder and mouse behavioral studies

**DOI:** 10.1093/hmg/ddu369

**Published:** 2014-07-15

**Authors:** Chie Shimamoto, Tetsuo Ohnishi, Motoko Maekawa, Akiko Watanabe, Hisako Ohba, Ryoichi Arai, Yoshimi Iwayama, Yasuko Hisano, Tomoko Toyota, Manabu Toyoshima, Katsuaki Suzuki, Yukihiko Shirayama, Kazuhiko Nakamura, Norio Mori, Yuji Owada, Tetsuyuki Kobayashi, Takeo Yoshikawa

**Affiliations:** 1 Department of Biological Sciences, Graduate School of Humanities and Sciences, Ochanomizu University, Tokyo 112-8610, Japan,; 2 Laboratory for Molecular Psychiatry, RIKEN Brain Science Institute, Saitama 351-0198, Japan,; 3 Division of Applied Biology, Faculty of Textile Science and Technology, Shinshu University, Nagano 386-8567, Japan,; 4 Department of Psychiatry, Hamamatsu University School of Medicine, Shizuoka 431-3192, Japan,; 5 Department of Psychiatry, Teikyo University Chiba Medical Center, Chiba 299-0111, Japan,; 6 Department of Neuropsychiatry, Hirosaki University Graduate School of Medicine, Aomori 036-8562, Japan and; 7 Department of Organ Anatomy, Yamaguchi University Graduate School of Medicine, Yamaguchi 755-8505, Japan

## Abstract

Disturbances of lipid metabolism have been implicated in psychiatric illnesses. We previously reported an association between the gene for fatty acid binding protein 7 (*FABP7*) and schizophrenia. Furthermore, we identified and reported several rare non-synonymous polymorphisms of the brain-expressed genes *FABP3*, *FABP5* and *FABP7* from schizophrenia and autism spectrum disorder (ASD), diseases known to part share genetic architecture. Here, we conducted further studies to better understand the contribution these genes make to the pathogenesis of schizophrenia and ASD. In postmortem brains, we detected altered mRNA expression levels of *FABP5* in schizophrenia, and of *FABP7* in ASD and altered *FABP5* in peripheral lymphocytes. Using a patient cohort, comprehensive mutation screening identified six missense and two frameshift variants from the three FABP genes. The two frameshift proteins, FABP3 E132fs and FABP7 N80fs, formed cellular aggregates and were unstable when expressed in cultured cells. The four missense mutants with predicted possible damaging outcomes showed no changes in intracellular localization. Examining ligand binding properties, FABP7 S86G and FABP7 V126L lost their preference for docosahexaenoic acid to linoleic acid. Finally, mice deficient in *Fabp3*, *Fabp5* and *Fabp7* were evaluated in a systematic behavioral test battery. The *Fabp3* knockout (KO) mice showed decreased social memory and novelty seeking, and *Fabp7* KO mice displayed hyperactive and anxiety-related phenotypes, while *Fabp5* KO mice showed no apparent phenotypes. In conclusion, disturbances in brain-expressed FABPs could represent an underlying disease mechanism in a proportion of schizophrenia and ASD sufferers.

## INTRODUCTION

Schizophrenia is a severe mental illness featuring three major symptomatic domains: positive symptoms (e.g. hallucinations and delusions), negative symptoms (e.g. affective flattening and avolition) and cognitive deficits (e.g. disorganized thought). It presents with a life-time prevalence of ∼1% worldwide (1). Autism spectrum disorder (ASD) is a relatively common childhood neurodevelopmental disorder, with an increasing incidence. The disease is characterized by severe impairment in social interaction and communication, and restricted and repetitive behavior (2). Multiple genetic factors, each with a small effect, environmental insults and an interaction of these factors, contribute to the pathology of these diseases. Increasing evidence supports the idea of shared genetic and environmental risk factors (3–6) between the two diseases.

Recently, fatty acids, particularly ω3 and ω6 polyunsaturated fatty acids (PUFAs) have attracted attention in the pathophysiology of schizophrenia and ASD. In schizophrenic patients, significantly lower levels of ω3 and ω6 PUFAs were observed in red blood cells (7,8), and in autistic patients, decreased ω3 PUFA was observed in plasma samples (9). The supplementation of PUFAs with psychotropic drugs improved symptoms in schizophrenia, although PUFAs alone are not sufficient as therapeutic agents (10,11). Our mouse study revealed that dietary PUFAs improve prepulse inhibition (PPI), which reflects sensorimotor gating function in the central nervous system, and is a typical endophenotype impaired in mental illnesses, including schizophrenia and ASD (12,13), although there are discrepancies among human studies (14–16).

As PUFAs are extremely lipophilic molecules, chaperone proteins called fatty acid binding proteins (FABPs) are needed for their intracellular trafficking. FABPs consist of at least 12 family members in mammals and 10 in humans (17). They are small 14–15 kDa intracellular proteins (18) and exist across species, from flies to mice and humans, with strong phylogenetic conservation (18). In the brain, three main types of *FABP*s, namely, *FABP3*, *FABP5* and *FABP7*, are expressed in specific cell populations, such as mature neurons, neural progenitor cells and neural stem/progenitor cells, respectively (19). Although their primary structures show strong homology, they exhibit specific fatty acid ligand preferences. FABP3 binds preferentially to ω6 PUFAs such as arachidonic acid (20:4) (20–22), FABP5 prefers saturated fatty acids such as stearic acid (18:0) and monounsaturated fatty acids such as oleic acid (OA, 18:1) (22–24), and FABP7 binds preferentially to ω3 PUFAs such as docosahexaenoic acid (DHA, 22:6) (21,22,25).

Our recent studies highlighted several lines of evidence showing that genetic variations in these brain-expressed *FABP* genes are directly involved in the pathogenesis of mental illnesses. First, we identified *Fabp7* using quantitative trait loci analysis in mice, as a gene that controls PPI (26). The *Fabp7* knockout (KO) mice displayed consistent decreases in PPI. Second, we detected a genetic association of *FABP7* with both schizophrenia and bipolar disorder (27). In another study, resequencing analysis of *FABP3*, *FABP5* and *FABP7* from an ASD cohort identified several rare non-synonymous polymorphisms, some of which were also seen in schizophrenia (28).

In this study, we performed a comprehensive analysis of brain-expressed *FABP* genes, extending our prior genetic studies (26–28). We aimed to elucidate possible roles for these genes in the pathophysiology of schizophrenia and ASD.

## RESULTS

### Transcript levels of *FABP3*, *FABP5* and *FABP7* in human postmortem brains and peripheral lymphocytes

**Figure 1.**
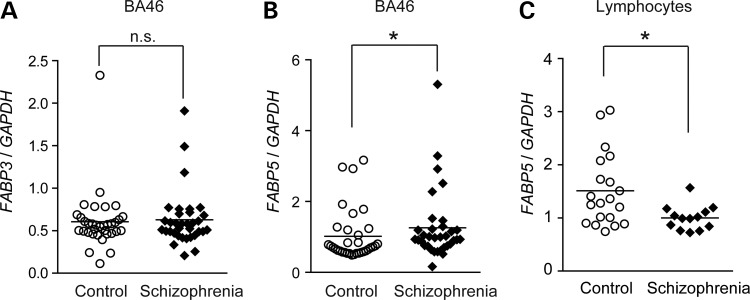
Transcript levels of *FABP3* and *5* in postmortem brains and lymphocytes from schizophrenic patients. (**A**, **B**) Transcript levels of *FABP3* (A) and *FABP5* (B) in postmortem brains (BA 46) from patients with schizophrenia (*n* = 34) and controls (*n* = 34). (**C**) Transcript levels of *FABP5* in lymphocytes from control (*n* = 20) and schizophrenia samples (*n* = 13). *GAPDH* was used as an internal control. Gene expression levels were evaluated using the two-tailed Mann–Whitney *U*-test. *P < 0.05.

Since our previous work showed upregulation of *FABP7* transcripts in the prefrontal cortex of schizophrenic postmortem brains (26), we extended the transcript expression studies to *FABP3* and *FABP5* using the same sample set. In schizophrenic postmortem brains (Brodmann's area 46: BA46, a region in the prefrontal cortex), we found elevated mRNA levels for *FABP5* (Fig. 1B), while those of *FABP3* were unchanged (Fig. 1A). We found no correlations between the expression levels of the *FABP*s and lifetime dosage of antipsychotics (Supplementary Material, Fig. S1A–C). Interestingly, we found that expression of *FABP5* and *FABP7* correlated tightly in both schizophrenia and control subjects (Supplementary Material, Fig. S1D and E), although the biological implications of this observation are unknown. In contrast, peripheral lymphocytes from drug-naive schizophrenics showed decreased *FABP5* expression (Fig. 1C). *FABP3* and *FABP7* were undetectable in peripheral lymphocytes.

**Figure 2.**
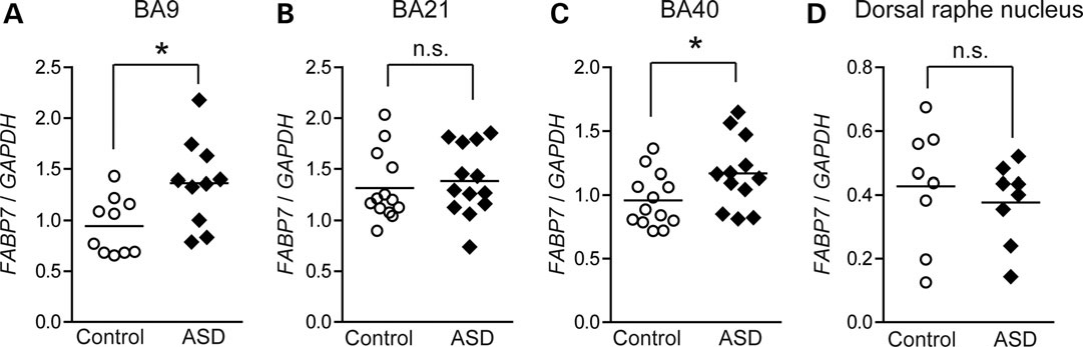
*FABP7* transcript levels in postmortem brains from patients with ASD. (**A**–**D**) *FABP7* transcript levels in postmortem brains from autistic patients and controls. We examined four regions of postmortem brains; BA 9: control (*n* = 10) and ASD samples (*n* = 10) (A), BA21: control (*n* = 13) and ASD samples (*n* = 13) (B), BA40: control (*n* = 13) and ASD samples (*n* = 12) (C) and the dorsal raphe nucleus: control (*n* = 8) and ASD samples (*n* = 8) (D). *GAPDH* was used as an internal control. The gene expression levels were evaluated as in Figure 1.

Next, we examined expression levels of the three *FABPs* in postmortem brain samples from autistic subjects (Fig. 2A–D and Supplementary Material, Fig. S2A–H). In this study, four different brain regions were tested: BA9 (a region of the frontal cortex), BA21 (a region of the temporal cortex), BA40 (a region of the parietal cortex) and the dorsal raphe nucleus. Interestingly, *FABP7* was upregulated in the BA9 and BA40 brain regions (Fig. 2A and C), but *FABP3* and *5* showed no significant changes in any brain regions (Supplementary Material, Fig. S2A–H).

These combined data imply aberrant expression of *FABP7* and *FABP5* genes in the brains and/or lymphocytes of schizophrenia and ASD sufferers, suggesting a pathophysiological role for these genes.

### Resequencing analyses of *FABP3*, *FABP5* and *FABP7*

Next, we extended our previous study and explored the possibility that rare variants of *FABP* genes encoding proteins with altered function may play a role in the pathogenesis of these two mental disorders (27,28). With this aim, we set out to resequence the protein-coding exons of these genes using a larger disease cohort consisting of 2097 schizophrenia patients and 316 ASD patients. We detected 16 variants in *FABP3*, *FABP5* and *FABP7* (Table 1 and Supplementary Material, Fig. S3). None of them satisfied statistical significance, and pooled analysis did not reveal any significance. Nine were novel and seven were previously reported. Of them, six were missense variants and two were frameshift-causing variants (Table 1). Next, we examined the frequency of these variants in 2170 control subjects, and found that the *FABP3* c.395delA (p.E132fs) and *FABP7* c.239delA (p.N80fs), c.256A>G (p.S86G) and c.376G>C (p.V126L) were exclusively seen in disease sufferers (Table 1). Where the patient's parents were available for examination, the detected non-synonymous and frameshift-causing variants were classified as non-*de novo* (Supplementary Material, Fig. S4). To date, no variants of *FABP* genes or in neighboring genes that may be in linkage disequilibrium with those on *FABP*s have been reported to be associated with schizophrenia and ASD (http://www.genome.gov/page.cfm?pageid=26525384#searchForm, threshold: *P* < 10^−5^). In addition, *FABP3*, *5* and *7* genes are not included in the copy number variation loci that have been reported to be associated with schizophrenia and ASD (29). Therefore, the detected variants might be population-specific (30).

**Table 1.** Polymorphisms detected in brain-expressed FABP genes

Gene	Exon	Nucleotide change	Amino acid change	db SNP^a^	SZ (2097)	Autism (316)	Control (2170)
*FABP3*	1	c.8A>G	p.D3G	rs17848124	47	5	41
	–	c.246+16_17delAG^b^	–	ss1026504480	–	1	–
	–	c.246_25delG^b^	–	ss974293059	1	–	–
	3	c.283C>T	Synonymous	rs138560753	5	–	–
	–	c.349-19A>G^b^	–	ss974293060	1	–	–
	–	c.349-13_14delGT^b^	–	ss1026504481	7	1	–
	4	c.395delA^b^	p.E132fs (p.Glu132GlyfsX71)	ss1026504456	1	0	0
*FABP5*	3	c.279A>G^b^	Synonymous	ss974293061	1	–	–
	3	c.331T>C^b^	Synonymous	ss974293062	2	1	–
	3	c.340G>C	p.G114R	rs75108814	3	1	1
	4	c.371A>G^b^	p.N124S	ss974293063	2	0	6
*FABP7*	2	c.182C>T	p.T61M	rs2279381	107	22	117
	2	c.239delA^b^	p.N80fs (p.Asn80Thrfsx27)	ss974293064	1	0	0
	3	c.256A>G	p.S86G	rs146649536	1	0	0
	–	c.348+21A>G	–	rs17848133	44	9	–
	4	c.376G>C	p.V126L	rs77153426	0	1	0

dbSNP, single nucleotide polymorphism database; FABP, fatty acid-binding protein; SZ, schizophrenia.

^a^The NCBI database (http://www.ncbi.nlm.nih.gov/) was searched for known SNPs.

^b^Novel variants.

### *In silico* predicted impact of variants on protein function

The G114 residue in FABP5 and the T61 and V126 residues in FABP7 are highly conserved among vertebrate species (Supplementary Material, Fig. S5), raising the possibility that substitutions at these residues could impact on biological function. The *in silico* tool-websites ‘SIFT’ (http://sift.bii.a-star.edu.sg/www/SIFT_BLink_submit.html), ‘Polyphen-2’ (http://genetics.bwh.harvard.edu/pph2/) and ‘Pmut’ (http://mmb2.pcb.ub.es:8080/PMut/) estimates the probable impact of an amino acid substitution on the structure and function of a protein. These algorithms predicted that the FABP5 G114R and FABP7 T61M, S86G and V126L mutations could ‘affect protein function’, be ‘probably damaging’ or ‘pathological’, and that the other two variants, FABP3 D3G and FABP5 N124S, are tolerated/benign/neutral (Table 2).

**Table 2.** Predicted functional effect of missense mutations using SIFT, PolyPhen-2 and Pmut

Gene	Amino acid change	Prediction of possible functional impact		
		SIFT^a^	PolyPhen-2^b^	Pmut^c^
*FABP3*	p.D3G	Tolerated	Benign	Neutral
*FABP5*	p.G114R	Affect protein function	Probably damaging	Pathological
	p.N124S	Tolerated	Benign	Neutral
*FABP7*	p.T61M	Tolerated	Probably damaging	Pathological
	p.S86G	Affect protein function	Benign	Neutral
	p.V126L	Tolerated	Benign	Pathological

^a^SIFT (http://sift.bii.a-star.edu.sg/www/SIFT_BLink_submit.html).

^b^PolyPhen-2 (http://genetics.bwh.harvard.edu/pph2/).

^c^Pmut (http://mmb2.pcb.ub.es:8080/PMut/).

**Figure 3.**
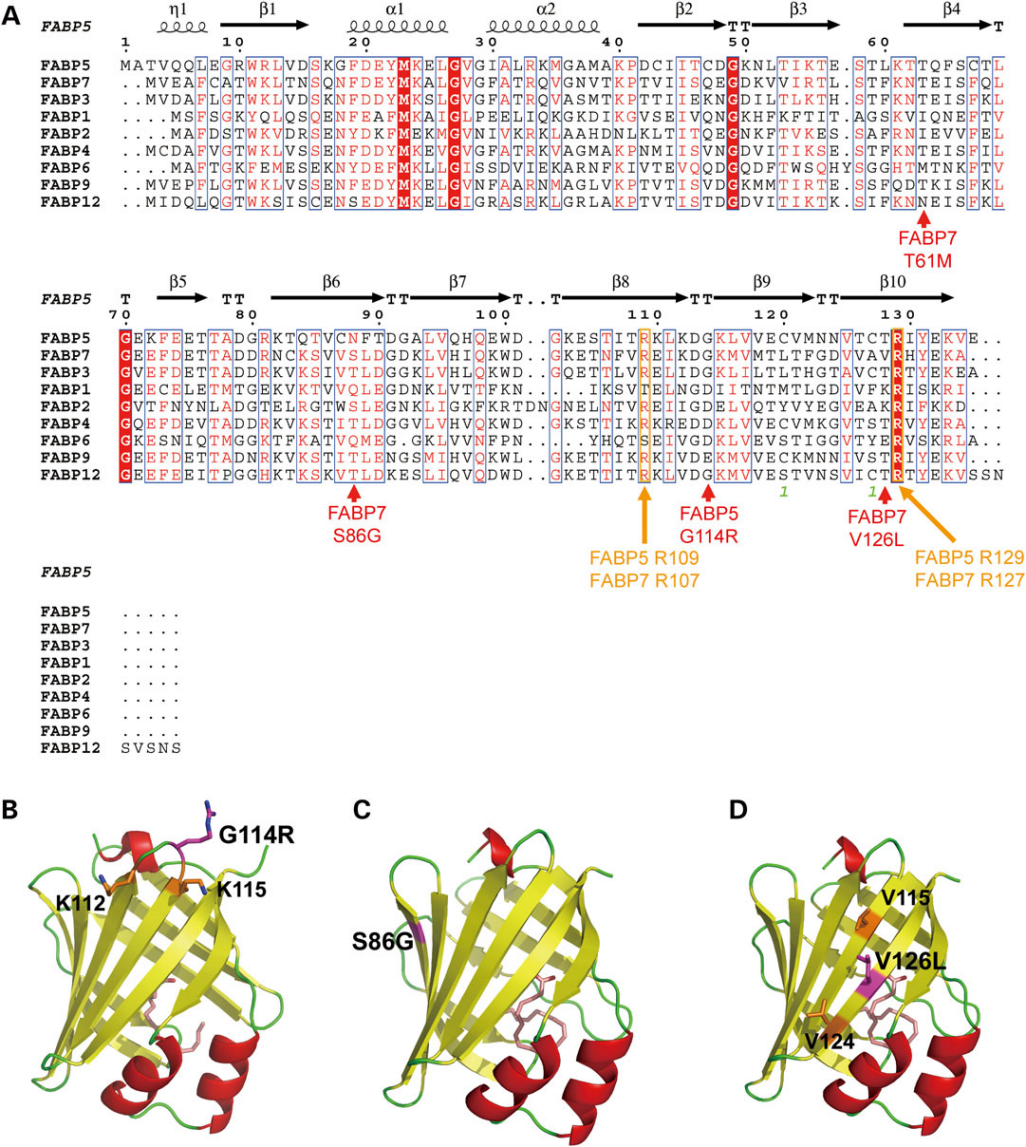
Possible impact of missense mutations on protein structure. (**A**) Amino acid sequence alignment of multiple human fatty acid binding proteins. The secondary structures of FABP5 (24) are schematically shown above the alignments using ESPript (31). The positions of mutations are indicated by arrows. (**B**) Model structure of the FABP5 G114R variant, generated by SWISS-MODEL (32,33), and based on the crystal structure of FABP5 (PDB ID, 1B56) (24). The bound palmitic acid is denoted in pale pink while the G114R residue is shown in magenta. The positively charged residues, K112 and K115, near G114R are denoted in orange. (**C**) Model structure of the FABP7 S86G variant, generated by SWISS-MODEL and based on the crystal structure of FABP7 (PDB ID, 1FDQ) (21). The bound DHA is denoted in pink and the site of S86G in magenta. (**D**) Model structure of the FABP7 V126L mutant, generated by SWISS-MODEL (32,33) and based on the crystal structure of FABP7 (PDB ID, 1FDQ) (21). The bound DHA is denoted in pink and the V126L residue in magenta. The hydrophobic residues, V115 and V124, near V126L are shown in orange. All graphics were made using PyMOL (DeLano Scientific, Palo Alto, CA, USA). The α-helices and the β-strands are shown in ribbon representation using the colors red and yellow, respectively.

Next, we evaluated the effects of the four missense variants with predicted functional damage, on the structural stability of the aberrant proteins, using secondary and tertiary structure models (Fig. 3A–D). The three-dimensional structures of the missense variants were generated by SWISS-MODEL (32,33), based on the crystal structures of FABP5 and FABP7 (21,24). The mutation site at G114R is located in the β8–β9 loop of FABP5 (24) (Fig. 3A and B). The new Arg produces an additional positive charge. This causes clustering of positive charges around Lys112 and Lys115, and may result in structural destabilization by electrostatic repulsion and undesired interactions with negatively-charged molecules. For FABP7 S86G, replacing a residue by Gly generally causes destabilization of the structure, since Gly lacks a side chain and therefore allows exceptionally wide conformation of the dihedral angle, compared with other amino acid residues. The missense site of S86G is located in the β-sheet of the FABP7 structure (21) (Fig. 3C), suggesting that this variation most likely destabilizes the β-sheet structure of FABP7. The V126L variant of FABP7 is located on the surface of the β-sheet exposed to solvent water (Fig. 3D). Generally, polar residues are essentially favorable when located on the surface of soluble globular proteins, in contrast to nonpolar residues. The nonpolar Val126 on the surface of FABP7 should be unfavorable by its nature and moreover, the substitution of Val126 by Leu would increase hydrophobicity. Therefore, the local hydrophobic patch consisting of Val115, Val124 and Leu126 on the surface of FABP7 L126 may promote undesirable interaction and protein aggregation. We previously reported on the possible effects of the FABP7 T61M variant on the protein structure (26).

**Figure 4.**
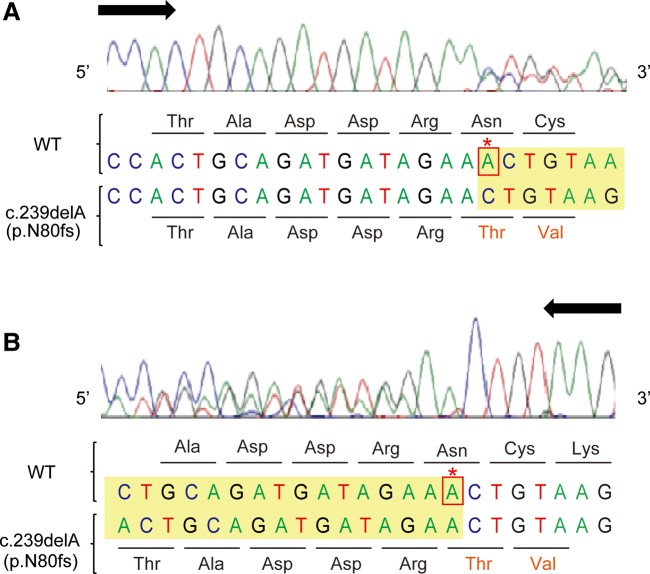
Direct sequencing of cDNA from the hair follicle cells of a patient carrying the *FABP7* c.239delA (p.N80fs) mutation. A cDNA spanning the *FABP7* c.239delA region was amplified by RT-PCR from hair follicle cells, and then direct-sequenced using forward (**A**) or reverse (**B**) primers. The asterisk indicates the deleted A base and frameshift starting point. Note that the sequencing peaks suggest that expression from the mutant allele is comparable to that of the WT allele in this patient.

The *FABP7* c.239delA (p.N80fs) variant, where a single ‘A’ base is deleted in exon 2, generates a premature termination codon in exon 3, raising the possibility that this mRNA is rapidly eliminated from cells by nonsense-mediated mRNA decay (NMD) system. However, this frameshift mRNA was expressed, to some extent, in the hair follicle cells from patients (Fig. 4). This is probably due to the close proximity of the premature termination codon to the final exon–exon junction (within 30 bp), preventing NMD (34). This is the case for the *FABP3* c.395delA (p.E132fs) mutation, where the deleted ‘A’ base is located in the same exon as the original termination codon, excluding the possibility of rapid degradation by NMD (34). Therefore, it is thought that substantial amounts of these frameshifted aberrant proteins are produced in cells.

Based on these results, we decided to investigate the functional consequences of the four missense variants, namely FABP5 G114R and FABP7 T61M/S86G/V126L and the two frameshift variants, FABP3 E132fs and FABP7 N80fs, by performing molecular and cellular analyses.

### Subcellular localization of FABP mutants

**Figure 5.**
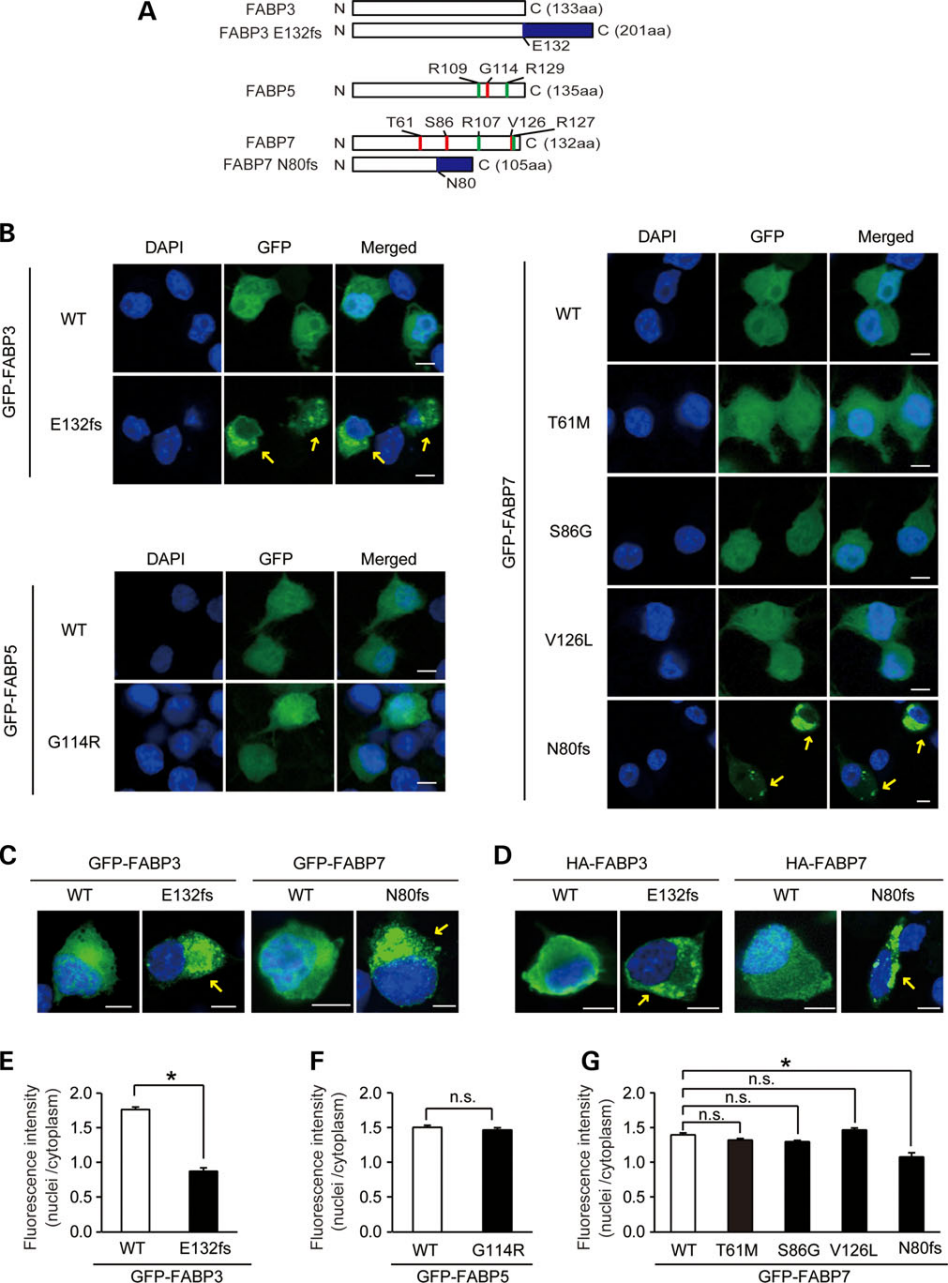
Aggregation of mutant proteins. (**A**) Schematic representation of mutant FABP proteins. Red lines indicate the position of the missense mutations, blue lines the position of frameshifts and green lines the position of binding pocket mutations used for the binding assay. (**B**) Subcellular localization of GFP-FABPs in Neuro2A cells. Nuclei were stained blue with DAPI. Yellow arrows show aggregate-like structures. Scale bar = 5 μm. (**C, D**) Subcellular localization of GFP-tagged (C) and HA-tagged (D) FABPs (WTs and frameshift mutants) in Neuro2A cells. Green signals denote exogenous FABPs. Nuclei were counterstained blue with DAPI. Scale bar = 5 μm. (**E–G**) The ratio of GFP fluorescence intensity of nuclei to cytoplasm for the GFP-FABP3s (E), GFP-FABP5s (F), GFP-FABP7s (G) expressed cells. Values are mean ± SEM of at least 100 GFP-positive cells. Data were evaluated using Mann Whitney's *U* test (E and F) or one-way ANOVA, followed by *post hoc* Bonferroni's multiple comparison test (G). *P < 0.0001.

FABPs play important roles in the transport of their insoluble ligands including free fatty acids, to various subcellular compartments such as the endoplasmic reticulum, mitochondria and nucleus. To investigate whether the subcellular localization of mutants differed from that of their wild-type (WT) counterparts, we transiently expressed N-terminal green fluorescent protein (GFP)-tagged FABPs in Neuro2A cells (a murine neuroblastoma cell-line). The positions of mutations within the FABP proteins are shown in Figure 5A. All the missense mutants localized to similar subcellular regions as the corresponding WT proteins (Fig. 5B). Interestingly, GFP-FABP3 E132fs and GFP-FABP7 N80fs formed aggregate-like structures outside of the nucleus (Fig. 5 B and C). We also examined the localization of frameshift mutants containing the much smaller HA (human influenza hemagglutinin) tag, since GFP shows a tendency to aggregate when overexpressed. Again, cellular aggregate-like structures were also observed in HA-FABP3 E132fs and HA-FABP7 N80fs-expressed cells (Fig. 5D), suggesting that these proteins are prone to aggregation.

Nuclear localization of GFP-FABP3 E132fs and GFP-FABP7 N80fs was much weaker compared with WTs (Fig. 5E and G), while there was no difference in the ratio of nuclear to cytoplasmic GFP fluorescence between the missense mutants and corresponding WTs (Fig. 5F and G). It is most likely that the frameshifted proteins are sequestered in the cytoplasmic compartment without entering the nucleus, due to of the formation of aggregates.

### Analyses of proteasome activity in the two frameshift mutants

**Figure 6.**
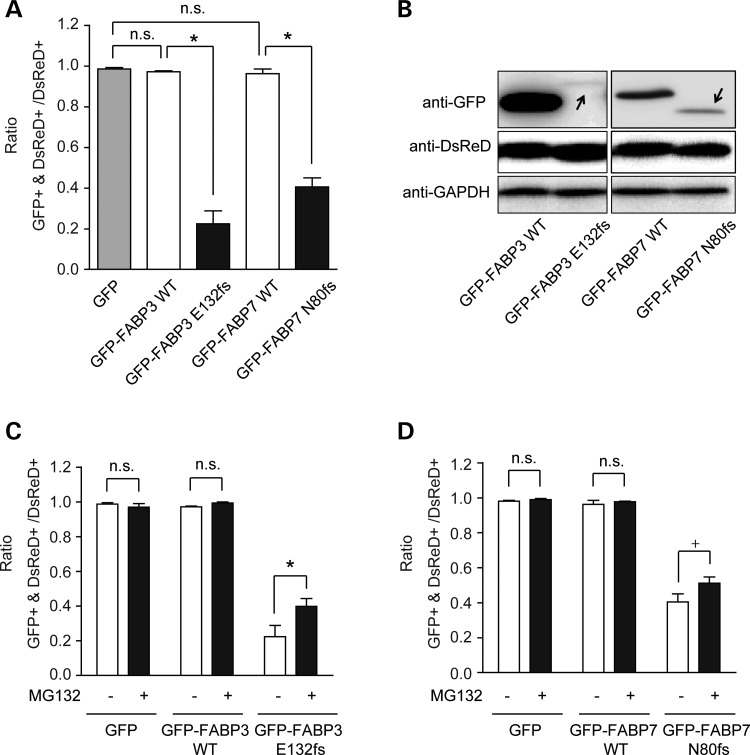
Instability of frameshift mutants. Neuro2A cells were co-transfected with a construct expressing GFP alone or GFP-FABPs, and a construct expressing DsRed. (**A**) The graph shows mean ± SEM for the ratio of GFP and DsRed-double-positive cells to DsRed only-positive cells (*n* = 100). The red signal of DsRed was used as a transfection marker. Data were evaluated using one-way ANOVA followed by *post hoc* Bonferroni's comparison test. *P < 0.001. (**B**) The expression levels of GFP-FABP proteins were examined by western blotting using anti-GFP antibody. The expression levels of DsRed and GAPDH were used as transfection and an endogenous (equal loading) controls, respectively. Arrows indicate the position for GFP-FABP frameshift mutant proteins. (**C, D**) The effects of proteasome inhibition on the abundance of GFP-FABP3 E132fs (C) and GFP-FABP7 N80fs (D). Twenty-four hours after co-transfection, cells were treated with or left without proteasome inhibitor, 1 μm MG132 for 8 h. Values are mean ± SEM for the ratio of GFP/DsRed-double-positive cells to DsRed only-positive cells (*n* = 100) obtained from three independent experiments. The effect of treatment was evaluated using one-way ANOVA followed by *post hoc* Bonferroni's comparison test. ^+^*P* < 0.1, *P < 0.05.

In the above experiments, we noticed a decrease in the number of GFP-FABP3 E132fs transfected cells and GFP-FABP7 N80fs transfected cells relative to cells transfected with WT constructs in transient expression experiments. Therefore, we attempted to quantitatively evaluate the expression of mutant proteins. To normalize transfection efficiency, cells were co-transfected with plasmid DNAs encoding GFP-FABPs and DsRed. Thirty-two hours after transfection, the ratio of GFP and DsRed-double positive cells to DsRed-only positive cells was calculated (Fig. 6A) and protein expression was evaluated by western blotting (Fig. 6B). The protein expression levels of GFP-FABP3 E132fs and GFP-FABP7 N80fs transfected cells were significantly lower than those of WT cells, even after normalization to DsRed.

Next, we examined whether these two frameshift mutants might be subject to proteasome degradation. Cells were co-transfected with plasmid DNAs encoding GFP-FABPs and DsRed. Twenty-four hours after transfection, the cells were treated with or without the proteasome inhibitor, MG132, for 8 h. The ratio of GFP and DsRed-double positive cells to DsRed only-positive cells was calculated. The results showed that the ratio of GFP-FABP3 E132fs-positive cells could be partially recovered by MG132 treatment (Fig. 6C), and that the ratio of FABP7 N80fs positive cells also showed a tendency to partial recovery (Fig. 6D). Taken together, it seems that active degradation of frameshift proteins by cellular proteolytic pathways, in other words, the proteasome system, can part explain the reduced expression levels of mutant proteins and the reduced number of frameshift FABP-transfected cells.

### Effects of missense variants on ligand binding potential

**Figure 7.**
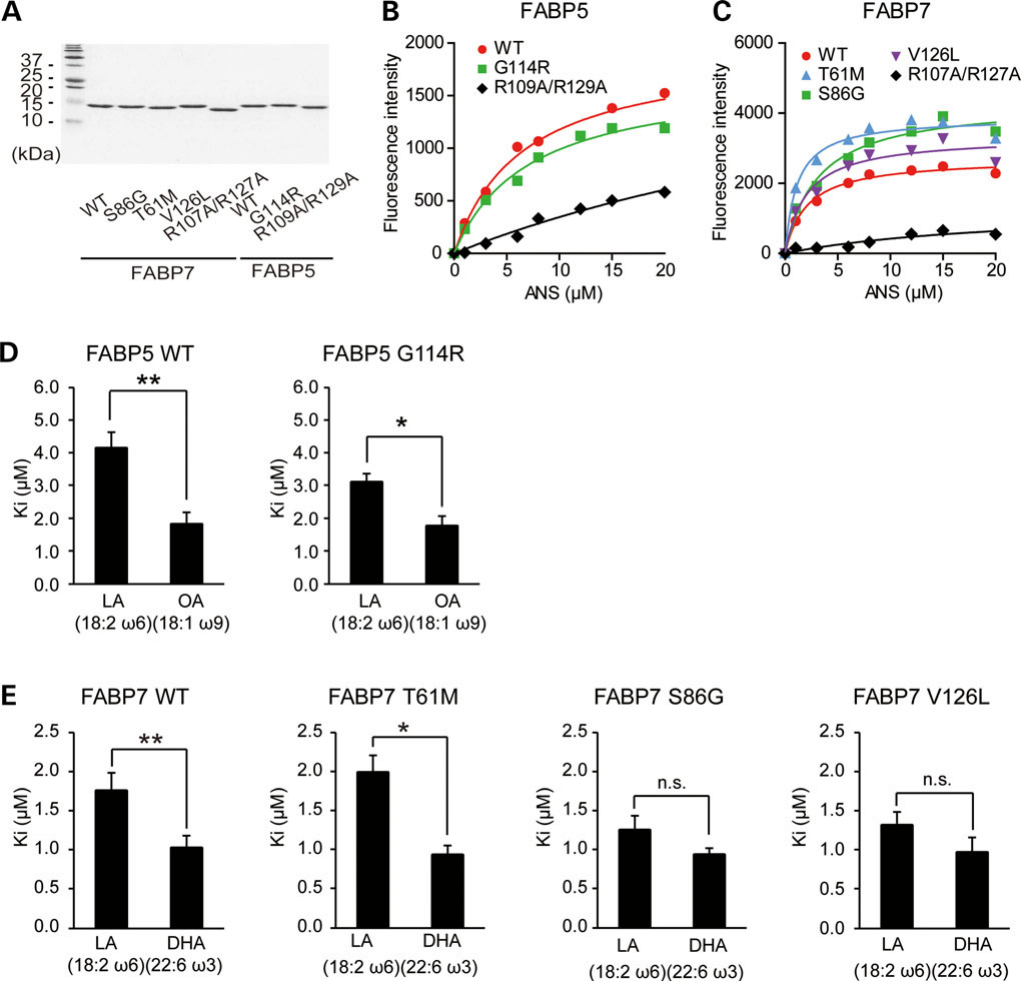
The effects of mutations on ligand binding potential. (**A**) Recombinant FABP proteins produced in *E. coli* and delipidated using Lipidex1000 were subjected to SDS-PAGE, followed by CBB staining. Each sample contained an equal loading of 1.5 μg of recombinant protein. (**B, C**) Increasing concentrations of ANS were incubated with 0.8 μm FABP5 (B) or FABP7 (C) and the intensity of fluorescence was recorded (excitation filter: 355 nm, and emission filter: 460 nm). Data shown are representative of duplicated titrations. Curves were fitted by non-linear regression analysis using GraphPad Prism software. (**D–G**) The *K*_i_ values obtained from ANS displacement assays. Representative model ligands for FABP5 are LA (D) and OA (E), and those for FABP7 are LA (F) and DHA (G). ANS was added to a 10-fold excess of FABPs, and an increasing concentration of fatty acid was added to the mixture. Curves were fitted by non-linear regression analysis using the GraphPad Prism software and *K*_i_ values are calculated using the tool-websites ‘The *K*_i_ calculator’ (http://sw16.im.med.umich.edu/software/calc_ki/). The *K*_i_ values were obtained from at least four independent experiments. (**H, I**) Ligand preferences of FABP5 (H) and FABP7 (I). The values represent mean ± SEM. Data were evaluated using unpaired *t*-test (D, E, H and I) or Dunnett's multiple comparisons test (F, G). *P < 0.01, ** *P* < 0.001, *n* = 4 (D, E, H), *n* = 6 (F, G, I).

To address the biochemical effects of missense variants on *FABP7* and *FABP5*, we determined the binding potential of mutant proteins to ligands. For this purpose, recombinant FABP proteins (FABP7 WT, S86G, T61M and V126L and FABP5 WT and G114R) were produced using bacteria, purified using glutathione sepharose beads and de-lipidated using a Lipidex1000 column. We also prepared double mutants for each of FABP5 (R109A/R129A) and FABP7 (R107A/R127A), as binding pocket-destructive mutants [negative controls for 1-anilinonaphthalene-8-sulfonic acid (ANS) titration assay]. Those four residues are highly conserved among the FABP (Fig. 3A and Supplementary Material, Fig. S5) and cellular retinoic acid-binding protein family (35), and previous studies have shown that these residues are important for the binding of fatty acids (21). We were able to purify these proteins to an almost homogeneous level (Fig. 7A).

First, we performed titration analyses of the various FABP affinities using ANS (Fig. 7B and C). ANS is a fluorescent dye that binds with high affinity to hydrophobic surfaces of proteins, such as FABPs (36–38). Compared with the binding dissociation constant (*K*_d_) values for WTs, those of the double mutants (FABP5 R109A/R129A and FABP7 R107A/R127A) were significantly higher, while those of the missense mutants detected in human samples were nearly identical to WT values (Table 3), demonstrating that the missense mutations do not affect the affinity to ANS.

**Table 3.** Binding parameters of FABP5 and FABP7 mutants for ANS

Protein	1, 8-ANS	
	*K*_d_ (μm)	Versus WT
FABP5		
WT	11.66 ± 3.13	
G114R	12.41 ± 1.77	*P* = 0.9938^a^
R109A/R129A	34.73 ± 9.31	*P* = 0.0353^a^
FABP7		
WT	5.54 ± 2.02	
T61M	2.85 ± 0.85	*P* = 0.6454^a^
S86G	6.00 ± 1.78	*P* = 0.9990^a^
V126L	4.34 ± 1.49	*P* = 0.9657^a^
R107A/R127A	21.81 ± 2.18	*P* < 0.0001^a^

Values are mean ± SEM of at least three independent experiments.

*K*_d_; dissociation constant.

*K*_i_; inhibition constant.

^a^One-way ANOVA with Dunnett's multiple comparison test.

Second, to characterize the binding potentials of the mutants to fatty acids, we evaluated inhibition constant (*K*_i_) values of the FABPs, using the ANS displacement assay. These assays were conducted using linoleic acid (LA) and OA for FABP5 mutants, and for FABP7 mutants, using LA and DHA, as representative model ligands [binding preference: FABP5; OA > LA (22, 23), FABP7; DHA > LA (21, 22)]. Competitive curves were obtained by adding increasing concentrations of fatty acids to preformed ANS–FABP complexes, and *K*_i_ values were calculated from at least four independent experiments (Table 4 and Fig. 7D and E). Figure 7D and E show the ligand preference of each FABP WT and mutant. None of the missense mutants showed significant differences in *K*_i_ values for fatty acids, as compared with WTs (Table 4). Both FABP5 G114R and FABP5 WT significantly preferred OA to LA (Fig. 7D). In contrast, while FABP7 T61M and FABP7 WT significantly preferred DHA to LA, FABP7 S86G and FABP7 V126L lost their preference for DHA over LA [e.g. a decreased (*K*_i_ for DHA/*K*_i_ for LA)] (Fig. 7E).

**Table 4.** Binding parameters of FABP5 and FABP7 mutants to representative natural ligands

Protein	LA		DHA		OA	
	*K*_i_ (μm)	Versus WT	*K*_i_ (μm)	Versus WT	*K*_i_ (μm)	Versus WT
FABP5						
WT	4.13 ± 0.50		–		1.81 ± 0.37	
G114R	3.09 ± 0.27	*P* = 0.1142^a^	–		1.76 ± 0.31	*P* = 0.9192^a^
FABP7						
WT	1.75 ± 0.23		1.02 ± 0.16		–	
T61M	1.99 ± 0.22	*P* = 0.7626^b^	0.93 ± 0.09	*P* = 0.9913^b^	–	
S86G	1.25 ± 0.19	*P* = 0.2255^b^	0.93 ± 0.12	*P* > 0.9999^b^	–	
V126L	1.31 ± 0.17	*P* = 0.3197^b^	0.96 ± 0.20	*P* > 0.9999^b^	–	

Values are mean ± SEM of at least four independent experiments.

*K*_d,_ dissociation constant.

*K*_i,_ inhibition constant.

^a^Unpaired *t-*test.

^b^One-way ANOVA with Dunnett's multiple comparison test.

### Behavioral analyses of *Fabp3*, *Fabp5* and *Fabp7* KO mice

For a more in-depth analysis of the biological functions of brain-expressed *FABP* genes and the consequences of a loss-of-function for these genes, we systematically characterized the behavior of *Fabps*-KO mice. As summarized in Table 5 and Supplementary Material, Table S1–3, we detected behavioral alterations in *Fabp3 and Fabp7* KO mice, suggesting critical roles for these genes in psychiatric illness phenotypes.

**Table 5.** Behavioral tests in Fabp3/5/7 KO mice

	*Fabp3 KO*	*Fabp5 KO*	*Fabp7 KO*
Home cage activity test			
Total locomotor activity	n.s.^a^	n.s.^a^	n.s.^a^
Open field test			
Total distance (cm)	n.s.^a^	n.s.^a^	n.s.^a^ (*P* = 0.0519)
Center time (%)	n.s.^a^	n.s.^a^	↓^a^ (*P* = 0.0025)
Y maze test			
Total entry number	n.s.^a^	n.s.^a^	n.s.^a^
Alternation (%)	n.s.^a^	n.s.^a^	n.s.^a^
Morris Water maze test			
Prove test			
Total distance (cm)	n.s. ^a^	n.s.^a^	n.s.^a^
Target area (%)	n.s.^a^	n.s.^a^	n.s.^a^
Opposite area (%)	n.s.^a^	n.s.^a^	n.s.^a^
Left area (%)	n.s.^a^	n.s.^a^	n.s.^a^
Right area (%)	n.s.^a^	↑^a^ (*P* = 0.0077)	n.s.^a^
Reversal prove test			
Total distance (cm)	n.s.^a^	n.s.^a^	n.s.^a^
Target area (%)	n.s.^a^	n.s.^a^	n.s.^a^
Opposite area (%)	n.s.^a^	n.s.^a^	n.s.^a^
Left area (%)	n.s.^a^	n.s.^a^	n.s.^a^
Right area (%)	n.s.^a^	n.s.^a^	n.s.^a^
Fear conditioning test			
Conditioning			
Freezing (%)	n.s.^a^	n.s.^a^	n.s.^a^
Contextual			
Freezing (%)	n.s.^a^	n.s.^a^	n.s.^a^
Elevated plus maze test			
Center (%)	n.s.^a^	n.s.^a^	n.s.^a^
Open (%)	n.s.^a^	n.s.^a^	n.s.^a^
Closed (%)	n.s.^a^	n.s.^a^	n.s.^a^
Light and dark box test			
Dark box (s)	n.s.^a^	n.s.^a^	n.s.^a^
Light box (s)	n.s.^a^	n.s.^a^	n.s.^a^
Ultrasonic vocalization			
Total frequency/5min	n.s.^b^	n.s.^b^	n.s.^b^
Forced-swim test			
Immobility time (s/5 min)	n.s.^a^	n.s.^a^	↓^a^ (*P* = 0.0376)
Tail suspension test			
Immobility (%)	n.s.^a^	n.s.^a^	n.s.^a^
Resident intruder test			
Contact time (s)	n.s.^a^	n.s.^a^	n.s.^a^ (*P* = 0.0639) [sniff/contact↓^a^ (*P* = 0.0078)]
Three-chamber test			
Session 1	n.s.^a^	n.s.^a^	n.s.^a^
Session 2	Familier ≒ stranger	n.s.^a^	n.s.^a^
PPI			
PP/P dB (%)	74/120 PP/P dB: n.s.^b^	74/120 PP/P dB: n.s.^b^	74/120 PP/P dB:↓^c^ (*P* < 0.05)
	78/120 PP/P dB: n.s.^b^	80/120 PP/P dB: n.s.^b^	78/120 PP/P dB:↓^c^ (*P* < 0.001)
	86/120 PP/P dB: n.s.^b^	86/120 PP/P dB: n.s.^b^	82/120 PP/P dB:↓^c^ (*P* < 0.001)
Administration of MK-801 (0.3 mg/kg)			
Post total locomotor activity/3 h	n.s.^b^	n.s.^b^	Challenge injection↑ (26)

ns; not significant.

^a^Mann–Whitney *U-*test.

^b^Two-way repeated-measures ANOVA (genotype effect; *P* > 0.05).

^c^Two-way repeated-measures ANOVA (genotype effect; *P* = 0.0004) followed by *post hoc* Bonferroni's comparison test.

**Figure 8.**
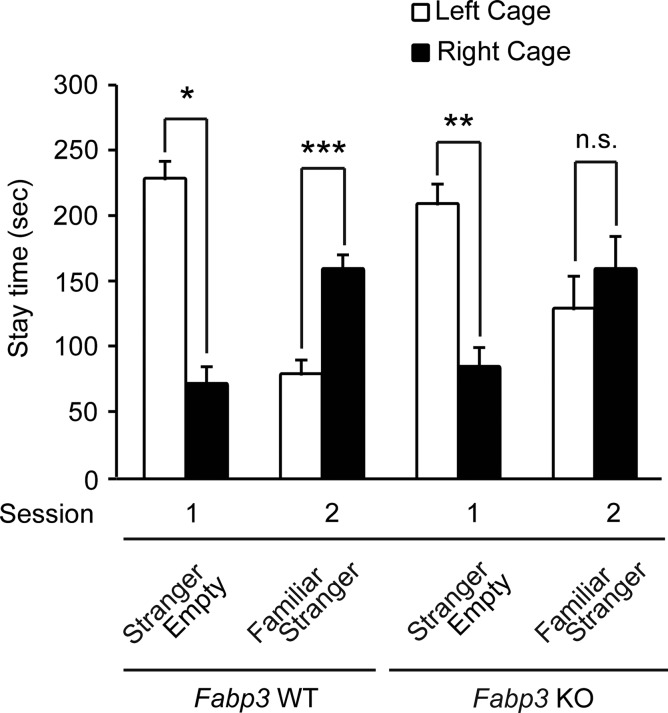
Social ability of *Fabp3* KO mice. Social affiliation and sociability were examined in Session 1. Mean stay time ± SEM in a chamber with a stranger (stranger) and in an opposite chamber (empty). Social memory and novelty seeking were examined in Session 2. Mean stay time ± SEM in the chamber with a mouse used in session 1 (familiar) and in the opposite chamber with a new unfamiliar mouse (stranger). Unlike WT controls (*n* = 13), KO mice (*n* = 10) failed to demonstrate a preference for social memory and novelty seeking. Data were evaluated using two-tailed Mann–Whitney *U-*test. *P < 0.01, **P < 0.001, ***P < 0.0001.

**Figure 9.**
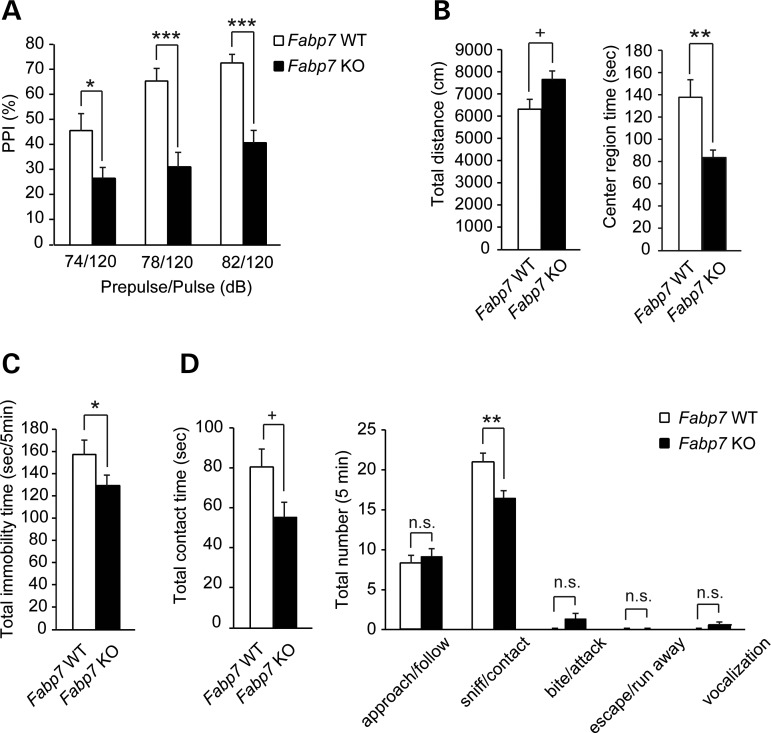
Characteristic behavioral alterations in *Fabp7* KO mice. (**A**) PPI test. The graph shows PPI (%) for WT (*n* = 13) and KO mice (*n* = 17). For statistical analyses of data, two-way repeated-measures ANOVA (genotype effect, *P* < 0.001) was followed by *post hoc* Bonferroni's comparison test. *P < 0.05, **P < 0.01, ***P < 0.001. (**B**) Open field test. Left graph shows total distances (cm) moved during 10 min and the right graph, the time spent in the center area (%) over a 10 min period for WT (*n* = 18) and KO (*n* = 20) mice. (**C**) Forced-swim test. The graph shows total immobility time (s) over a 5 min period for WT (*n* = 15) and KO (*n* = 18) mice. (**D**) Resident intruder test. Left graph shows total contact time (s) and the right graph, the total number of contacts for each behavior type in residents (WT and KO mice, *n* = 10 each) towards intruders, over a 10 min period. Data were analyzed using two-tailed Mann–Whitney *U-*test (B–E). Values are mean ± SEM. ^+^*P* < 0.1, *P < 0.05, **P < 0.01.

*Fabp3* KO mice exhibited decreased performance in social motivation and novelty seeking compared with WT mice, in the three-chamber test, although some researchers have cautioned about a potential risk for inappropriate interpretation of the data obtained from the three-chamber test (39). Unlike WT mice, *Fabp3* KO mice showed no preference for a newly introduced mouse (stranger) (Table 5, Supplementary Material, Table S1 and Fig. 8). There were no apparent changes in any other tests (Supplementary Material, Figs S6–9). The *Fabp5* KO mice displayed no apparent phenotypic changes, as shown in Table 5, Supplementary Material, Table S2 and Supplementary Material, Figs. S10–13. Current results for *Fabp7* KO mice replicated our previous data, showing decreased PPI (Table 5, Supplementary Material, Table S3 and Fig. 9A) (26), as well as a new discovery that male *Fabp7* KO mice exhibited hyperactivity and an anxiety-related trait when compared with WT: (1) slightly increased total distances (*P* = 0.0515) and decreased time spent in the center area in the open field test (*P* = 0.0025) (Table 5, Supplementary Material, Table S3 and Fig. 9B), (2) decreased immobility time in the forced-swim test (*P* *=* 0.0376) (Table 5, Supplementary Material, Table S3 and Fig. 9C), (3) slightly decreased total contact time (*P* = 0.0639) and a decreased sniffing/contact in the resident intruder test (*P* = 0.0078) (Table 5, Supplementary Material, Table S3 and Fig. 9D). The results of additional behavioral analyses in these mice are shown in Table 5 and Supplementary Material, Table S3 and Figs S14–16. These results support data proposing a role for *Fabp7* in sensorimotor gating function and emotional control (40).

## DISCUSSION

In this study, we performed comprehensive analyses of *FABP7*, *FABP5* and *FABP3,* in the context of psychiatric illnesses, particularly schizophrenia and ASD. By resequencing relevant exons, we identified 16 different variants, of which, nine were novel and seven had been previously reported. Importantly, six missense and two frameshift-causing variants were included, and of these, *FABP3* c.395delA (p.E132fs) and *FABP7* c.239delA (p.N80fs), c.256A>G (p.S86G) and c.376G>C (p.V126L) were seen exclusively in disease subjects. Although these variants were rare, we still investigated their functional consequences based on recent evidence proposing a polygenic burden of rare disruptive mutations in schizophrenia and neurodevelopmental disorders (41). When overexpressed in Neuro2A cells, FABP3 E132fs and FABP7 N80fs formed aggregate-like structures in the cytoplasm without entering the nucleus, implying elimination of transcription-regulatory function for these proteins since they cannot deliver ligands to nuclear factors (18,42). Furthermore, these frameshift proteins appeared instable in the cell. Our results may suggest the following possibilities: (i) frameshift mutant proteins aggregate and co-segregate with other proteins, as is the case with some aggresomes (43), and these misassembled complexes result in a toxic gain of function, and (ii) the frameshift mutant proteins may undergo a loss-of-function, due to removal by positive degradation or because the aberrant proteins simply lose the ability to bind natural ligands. Of note, two patients, one of whom carried the *FABP3* frameshift mutation (c.395delA) and one with the *FABP7* frameshift mutation (c.239delA), suffered from hyperlipidemia (Supplementary Material, Note).

Examining ligand binding properties, FABP7 S86G and FABP7 V126L, which were detected exclusively in schizophrenia subjects and an ASD subject, respectively, lost preference for DHA to LA. Considering that ω3, specifically DHA, showed a greater reduction relative to ω6 PUFA in the subset of schizophrenia patients who displayed lowered levels of PUFA (44), our findings imply that unbalanced utilization of essential fatty acids may contribute to the pathogenesis of schizophrenia and ASD.

In order to examine the biological roles of the brain-expressed *FABP* genes, we characterized behavioral phenotypes of the relevant KO mice. We found that *Fabp7* KO mice were hyperactive with an anxiety-related trait, based on a battery of behavioral tests. FABP7 exhibited a higher affinity for ω3 long chain PUFAs when compared with FABP3 and FABP5 (21), and is expressed in astrocytes, neural stem cells and oligodendrocyte progenitor cells of the developing brain (40,45,46). It is conceivable that the observed emotion-related phenotypes may stem from disturbed metabolism of those PUFAs and concomitant impaired neurodevelopment. Notably, Owada *et al.* (40) found that DHA is significantly decreased in *Fabp7* KO mice at the postnatal stage, and we reported decreased neurogenesis in the hippocampus of null mice (26).

There is ample evidence linking FABP7 to the *N*-methyl-d-aspartate (NMDA) receptor: (i) DHA potentiates NMDA-induced responses (47), (ii) *Fabp7* KO mice lack the ability to modulate NMDA receptor activation through DHA, in hippocampal neurons (40) and (iii) *Fabp7* KO mice evoke a greater behavioral response, compared with WT animals to repeated administration of the non-competitive NMDA receptor antagonist, MK-801 (26). In this study, untreated *Fabp7* KO mice displayed a hyperactive phenotype. It is of note that reduced NMDA receptor function is implicated in the pathophysiology of ASD, as well as schizophrenia (48–50). Since deficits in PPI are not specific to schizophrenia or ASD, but also shown in multiple neuropsychiatric disorders including bipolar disorder (51), a certain form of attention deficit hyperactivity disorder (52) and obsessive-compulsive disorder (OCD) (53), it would be interesting to explore the possibility that *FABP7* could be a common contributor to all these disorders.

We found that *Fabp3* KO mice exhibited decreased performance in social motivation and novelty seeking compared with WT mice, in the three-chamber tests. It would not be unreasonable to conclude that a functional deficiency of *FABP3* in humans may lead to cognitive dysfunction. In keeping with this conclusion, a *FABP3* frameshift mutation, which could potentially result in a loss of gene function, was found in a patient with residual schizophrenia (Supplementary Material, Note). Importantly, Fabp3 interacts directly with the dopamine D2 receptor long isoform (D2RL) (54) and *Fabp3* KO mice exhibit dopamine D2 receptor dysfunction (55).

In the current study, *Fabp5* KO mice showed no apparent disease phenotypes. This result raises the possibility that a loss-of-function of the *FABP5* gene itself has little effect, at least under normal nutritional conditions. Additionally, our results suggest that other *Fabp*s may compensate for *Fabp5* deficits in mice, given the compensatory interactions among *Fabp*s (56–59).

We also showed disturbances in the expression of brain-expressed *FABP* genes in patients suffering from psychiatric illnesses. First, we found elevated *FABP5* mRNA in the prefrontal cortex of schizophrenia postmortem brains. We have previously reported similar results for *FABP7* (26). When autism postmortem brains were analyzed, *FABP7* mRNA was elevated in the frontal cortex, as in schizophrenia, and also in the parietal cortex. *FABP7* may act in a disease pathway shared between schizophrenia and ASD. In contrast to postmortem brains, *FABP5* levels were lower in drug-naive schizophrenia derived peripheral lymphocytes. Since our study using mice showed no apparent effect for antipsychotic administration on the expression levels of *Fabp3* (Supplementary Material, Fig. S17A), *Fabp5* (Supplementary Material, Fig. S17B) or *Fabp7* (26) in the frontal cortex, it is probable that the differences in *FABP5* expression between brain and lymphocyte samples were not due to the effects of antipsychotics. A reverse pattern of differential concentrations of biomolecules between brain and peripheral blood samples has sometimes been observed. For example, it is reported that schizophrenic patients show lower concentrations DHA in red blood cells and higher concentrations DHA in cerebrospinal fluid (60). It would be of interest to determine whether expression levels of *FABP5* correlate with levels of DHA or other fatty acids. If so, it would be feasible to investigate the use of lowered FABP5 protein and DHA levels in the blood as biological markers for schizophrenia.

Current intriguing questions would focus on how upregulation of *FABP5* and *FABP7* in schizophrenia derived postmortem brains and upregulation of *FABP7* in autism derived postmortem brains are associated with disease. It would also be interesting to elucidate how disruption of *Fabp7* induces schizophrenia- and ASD-like, as well as emotion-related phenotypes in mice. Expression of the *FABP*s/*Fabps* are spatio-temporally controlled in the brain, with *Fabp7* and *Fabp5* abundantly expressed during neurodevelopment, in contrast to *Fabp3*, where expression plateaus in adulthood (45). We speculate that a proportion of schizophrenic and autistic patients may suffer from disturbances in either the use or metabolism of fatty acids, especially ω3 PUFAs, in early development, and that the upregulation of these genes in adulthood reflects an enduring compensatory mechanism, reminiscent of fetal programming or the developmental origins of health and disease (61,62).

Genetically, we identified rare variants by exon resequencing, and examined their functional consequences. All *FABP* family genes contain a canonical TATA box, followed by a conserved gene structure. However, the tissue-specific and developmentally regulated control of *FABP* gene expression is poorly understood, suggesting the presence of unidentified regulatory elements (26,63). Therefore, we cannot exclude a possible effect of sequence variants on genomic stretches, and nor can a broader genetic impact on schizophrenia and ASD be excluded.

In the present study, we have identified multiple rare variants in brain-expressed *FABP* genes. However, it still remains unknown whether these mutations are really causative in psychiatric patients. One of the means to approach this issue would be to examine a very large cohort like Psychiatric Genomics Consortium (PGC), and to test for polygenic inheritance from lower-frequency variants by using GWAS summary association statistics (64). Targeted exome sequencing using a large cohort would be also helpful for studying multiple genes as a group.

In summary, this study suggests that dysregulation or dysfunction of brain-expressed *FABPs* could represent a disease pathway linked to the lipid metabolic abnormality theory of schizophrenia and ASD. Further research, including the examination of other ethnic populations, is needed to utilize the lipid system in the generation of preventive and more beneficial therapies for patients with schizophrenia and ASD.

## MATERIALS AND METHODS

### Ethical issues

This study was approved by the Ethics Committees of RIKEN and all participating institutes, and conducted according to the principles expressed in the Declaration of Helsinki. All controls and patients, and their parents, if participants were <20 years old, gave informed, written consent to participate in the study after receiving a full explanation of the study protocols and objectives.

### Human postmortem brains

Demographic data on schizophrenia and control postmortem brain tissues (from the Stanley Medical Research Institute, Bethesda, MD, USA) have been described elsewhere (65).

The brain regions and the demographic data of autistic and control postmortem brain tissues (from the Maryland Psychiatric Research Center, Baltimore, MD, USA) were as follows: BA9 (ASD; 7 men, 3 women, mean age 13.5 ± 5.9 years, controls; 7 men, 3 women, mean age 13.7 ± 5.7 years), BA 21 (ASD; 10 men, 4 women, mean age 12.2 ± 5.6 years, controls; 10 men, 4 women, mean age 12.4 ± 5.4), BA 40 (ASD; 10 men, 4 women, mean age 12.2 ± 5.6 years, controls; 8–9 men, 4 women, mean age 12.8 ± 5.4 years), and dorsal raphe nucleus (ASD; 5 men, 3 women, mean age 15.5 ± 4.6 years, controls; 6 men, 2 women, mean age 15.8 ± 4.2 years).

### Real-time quantitative RT-PCR

Total RNA was extracted from mouse and human brain samples and human peripheral lymphocytes using miRNeasy Mini Kit (Qiagen, Valencia, CA, USA) and single stranded cDNA was synthesized using High Capacity RNA-to-cDNA Master Mix (Applied Biosystems, Foster City, CA, USA) and SuperScript VILO Master Mix (Invitrogen, Carlsbad, CA, USA). The mRNA levels were determined by real-time quantitative polymerase chain reaction (PCR) as already described (65). The *GAPDH*/*Gapdh* was chosen as an internal control (Applied Biosystems).

### Subjects

For the expression studies using lymphocytes, 14 drug-naive schizophrenia patients (7 men and 7 women; mean age 31.2 ± 12.3 years) and 20 controls (11 men and 9 women; mean age 36.0 ± 10.7 years) were used.

For the resequencing analysis, a total of 316 autistic patients of Japanese descent (262 men, 53 women and 1 unknown; aged between 3 and 32 years) were used. The diagnosis of ASD was made using the Diagnostic and Statistical Manual of Mental Disorders-IV (DSM-IV), followed by the ASD Diagnostic Interview-Revised criteria (66). Assessments were carried out by trained clinicians at Hamamatsu University and RIKEN. The diagnosis of schizophrenia was made by at least two experienced psychiatrists, using DSM-IV criteria: 2097 schizophrenics (1165 men and 929 women; mean age 41.4 ± 7.2 years) took part in this study along with 2170 controls (889 men and 1281 women; mean age 42.4 ± 14.2 years) who were deemed free of mental disorders during brief interviews with psychiatrists.

### Resequencing analysis of *FABP* genes in human subjects

We performed resequencing analysis of *FABP3*, *FABP5* and *FABP7* in human subjects. Protein-coding regions and exon/intron boundaries of the three genes were screened for polymorphisms by direct sequencing of PCR products (28). Detailed information is available upon request. Identified variants have been deposited in the NCBI database (http://www.ncbi.nlm.nih.gov/SNP/) and have been assigned the tentative IDs.

### *In silico* prediction of variants impact on protein function

We predicted the possible impact of amino acid substitutions on the structure and function of proteins using the tool-websites ‘SIFT’ (http://sift.bii.a-star.edu.sg/www/SIFT_BLink_submit.html), ‘Polyphen-2’ (http://genetics.bwh.harvard.edu/pph2/) and ‘Pmut’ (http://mmb2.pcb.ub.es:8080/PMut/).

The structural stability of proteins was also estimated using secondary and three-dimensional models. Three-dimensional models of the missense variants, based on the crystal structures of FABP5 and FABP7 (21,24), were generated by SWISS-MODEL (http://swissmodel.expasy.org/) (32,33).

### Construction of plasmids

Human *FABP3*, *FABP5* and *FABP7* cDNAs covering the open reading frame and untranslated regions were generated by PCR, using Marathon-Ready cDNA (derived from human embryonic brain; Clontech, Mountain View, CA, USA) and the following primer sets; *FABP3* forward: 5′-CCTGCTCTCTTGTAGCTTCTCTCA-3′, *FABP3* reverse: 5′-TGAGGCAATGTGGTGCTGAGTCGA-3′, *FABP5* forward: 5′-TTATAAAGCAGCCGCCGGCGCCGGGT-3′, *FABP5* reverse: 5′-GAGAATGACCAAGCTCAGTTCAATGAGC-3′, *FABP7* forward: 5′-AGGAGCTGCTTGCTGAGGTGTAAA-3′, and *FABP7* reverse: 5′-GGATAGCACTGAGACTTGAGGAAAC-3′. Each amplified cDNA was cloned into a pcDNA3 vector (Invitrogen). All mutants, excluding the frameshift constructs were derived from general site-directed mutagenesis. PCR fragments including frameshift mutants were amplified from the pcDNA3-FABP series DNA using primers with restriction enzyme sites at the 5′ terminal, and then cloned into the mammalian expression vector pCMV-HA (Clontech) using *Sal*I/*Xho*I sites, and into the pAcGFP1-C1 vector (Clontech) expressing N-terminal GFP tagged protein using, *Xho*I/*Bam*HI sites and *Sal*I/*Not*I sites for *FABP5/7* and *FABP3*, respectively. The same fragments were also inserted into the bacterial expression vector, pGEX-6P-3 （GE Healthcare Life Sciences, Tokyo, Japan）using *Xho*I/*Bam*HI for *FABP5* and *7* and *Sal*I/*Not*I sites for *FABP3*. The sequence validity of all generated plasmids was checked by DNA sequencing.

### Cells and transfection

The mouse neuroblastoma cell-line Neuro2A was maintained in Minimum Essential Media, supplemented with 1% non-essential amino acids, 10% fetal bovine serum, 100 U/ml penicillin and 100 U/ml streptomycin. Cells were transfected using Lipofectamine 2000 (Invitrogen) according to the manufacturer's protocol.

### Analysis of subcellular localization

Neuro2A cells grown in 8-well chamber slides (Nunc, Thermo Fishers, Rochester, NY, USA) were transiently transfected with plasmid DNA encoding GFP-FABPs. Forty-eight hours after transfection, cells were fixed with 4% paraformaldehyde in PBS, for 30 min on ice. After fixation, cells were permeabilized with 0.2% Triton X-100 in PBS for 15 min at room temperature (RT). Cells transiently expressing HA-FABPs were incubated with blocking solution (5% normal goat serum in PBS containing 0.1% Triton X-100) for 1 h at RT, incubated with antibodies against HA (clone 3F10, rat monoclonal; Roche Diagnostics, Mannheim, Germany) to a final concentration of 0.25 μg/ml in blocking buffer, overnight at 4°C. Slides were then washed three times with PBS for 5 min, incubated with Alexa Fluor 488–conjugated secondary antibodies (Invitrogen) in blocking buffer, at a final concentration of 5 μg/ml for 1 h at RT, and then washed three times with PBS for 5 min.

Cells were stained with 4′, 6-diamidino-2-phenylindole (DAPI) and mounted with PermaFluor Mountant (Thermo Fisher Scientific, Yokohama, Japan). The samples were scanned using a confocal laser scanning microscope system (FV1000D; Olympus, Tokyo, Japan) and analyzed with Meta-Morph software (Molecular Devices, Tokyo, Japan). Data were statistically evaluated by one-way analyses of variance (ANOVA), followed by *post hoc* Dunn's multiple comparison test. *P* < 0.05 was considered significant.

### Western blot analysis

The transfected cells were transferred to a microcentrifuge tube, and the pellets collected after centrifugation. Cells were lysed in ice-cold lysis buffer [50 mm Tris–HCl (pH 7.5), 150 mm NaCl, 0.5 mm ethylene glycol tetraacetic acid, 0.5% sodium dodecyl sulfate (SDS) and 0.5% Triton X-100] containing protease inhibitors [0.5 mm phenylmethylsulfonyl ﬂuoride (PMSF), 12 μg/ml aprotinin] by sonication and centrifuged at 4°C for 10 min at maximum speed. The supernatants were assayed for total protein concentration with the Dc Protein assay kit (Bio-Rad, Hercules, CA, USA), and concentrations were equalized using lysis buffer. Supernatants were diluted with 4× sample buffer [0.25m Tris–HCl (pH 6.8), 8% SDS, 20% sucrose, 0.02% bromophenol blue (BPB), 20% 2-mercaptoethanol] and boiled for 5 min. Proteins were separated on 15% SDS-polyacrylamide gels and transferred to polyvinylidene diﬂuoride membranes. The membranes were incubated for at least 1 h in blocking solution [5% skimmed milk in phosphate-buffered saline with 0.05% Tween 20 (PBS-T) and then incubated with primary antibody in blocking solution. Living Colors DsRed monoclonal antibody (Clontech) was used at a final concentration of 0.5 μg/ml for 2 h, *anti-GFP* monoclonal antibody (clone *1E4*; MBL, Nagoya, Japan) at a final concentration of 0.25 μg/ml for 1 h and anti-GAPDH antibody (V-18; Santa Cruz Biotechnology, Santa Cruz, CA, USA) at a final concentration of 0.4 μg/ml for 1 h. Next, membranes were washed three times with PBS-T for 5 min, incubated for 1 h with secondary antibody [horseradish peroxidase-conjugated anti-mouse IgG (GE Healthcare Life Sciences) or horseradish peroxidase-conjugated anti-rabbit IgG (Santa Cruz Biotechnology)], washed three times with PBS-T for 5 min, and detected using Western Blot Detection Reagent (Millipore, Billerica, MA, USA), according to the manufacturer's instructions. Chemiluminescent signals were captured by an image analyzer LAS-1000 (Fujiﬁlm, Tokyo, Japan).

### Cotransfection assay

Neuro2A cells grown in an 8-well chamber slides (Nunc) were transiently co-transfected with plasmids encoding GFP-FABPs and DsRed in a 1:1 ratio. Thirty-two hours after transfection, cells were fixed with 4% paraformaldehyde in PBS for 30 min on ice. Fixed cells were permeabilized with 0.2% Triton X-100 in PBS for 15 min at RT, then washed again with PBS. Cells were stained with DAPI and mounted with PermaFluor Mountant (Thermo Fisher Scientific). The samples were scanned and DsRed-positive cells and GFP/DsRed-double-positive cells were counted using Meta-Morph software (Molecular Devices). For proteasome inhibition analyses, 8 h before fixation, cells were treated with 1 μm MG132 (Calbiochem, San Diego, CA, USA). Data were evaluated using one-way ANOVA, followed by *post hoc* Bonferroni's comparison test.

### Recombinant FABP proteins

All WT and mutant cDNAs encoding human FABPs were subcloned into the bacterial expression vector pGEX-6P-3 (GE Healthcare Life Sciences), and transformed into *Escherichia coli* BL21 (DE3). The *E. coli* cells were grown at 37°C and induced at an OD_600_ of 1.0, with 0.1 mm isopropyl thiogalactoside for 6 h at 20°C. The harvested cells were lysed by sonication in sonication buffer [50 mm Tris–HCl (pH 8.0), 150 mm NaCl, 1 mm ethylenediamine tetraacetic acid (EDTA), 1 mm PMSF, 1 mm dithiothreitol (DTT), 1 mg/ml lysozyme]. Triton X-100 was added to cell lysates at a final concentration of 1%. The lysates were kept for 20 min at 4°C, with occasional gentle mixing. The centrifuged supernatant was added to a 50% slurry of Glutathione Sepharose 4B (GE Healthcare Life Sciences) pre-equilibrated with PBS (pH 7.3) and incubated overnight. After extensive washing with PBS (pH 7.3) containing 1 mm DTT, GST-fusion proteins captured on the beads were treated for 4 h at 4°C with PreScission protease (GE Healthcare Life Sciences), which cleaves between the Gln and Gly residues of the recognition sequence of LeuGluValLeuPheGlnGlyPro, in cleavage buffer [50 mm Tris–HCl (pH 7.5), 150 mm NaCl, 1 mm DTT, 1 mm EDTA]. The resultant FABP recombinant proteins lack the GST portion and harbor additional 5 or 11 amino acids derived from the vector at the N-terminal. The mixture was centrifuged at 500×*g* for 5 min. The supernatant containing FABPs was concentrated with Amicon Ultra-4 centrifugal filter devices (Millipore) with a 10 kDa molecular weight cutoff and delipidated by incubation with Lipidex-1000 (PerkinElmer, Waltham, MA, USA) as reported previously (67,68). Protein purity was confirmed by Coomassie brilliant blue (CBB) staining following SDS-PAGE, and the protein concentrations were determined using the Dc Protein assay kit (Bio-Rad).

### Binding assay

The fluorescent probe, 1-anilinonaphthalene-8-sulfonic acid (ANS) and free fatty acids, LA (18:2 ω6), DHA (22:6 ω3) and OA (18:1 ω9), were purchased from Cayman Chemical (Ann Arbor, MI, USA). Binding assays using ANS were based on a procedure described elsewhere (23,69,70). Titrations of FABPs with ANS were studied by measuring changes in fluorescence during titrations of ANS into a fixed concentration of FABPs. The mixtures of ANS and each FABP (0.8 μm) in 50 μl buffer (10 mm potassium phosphate, 1.62 mm disodium hydrogen phosphate 2.74 mm NaCl and 40.54 mm KCl, pH 7.4) were kept at 25°C for 3 min in the dark. Fluorescence was measured using a multi-label counter (excitation at 355 nm, emission at 460 nm; Wallac 1420 ARVO MX-2, Perkin Elmer). The fluorescence data were analyzed and the dissociation constant (*K*_d_) values were calculated by non-linear regression analysis using GraphPad Prism software (version 5.04, GraphPad Software, San Diego, CA, USA).

ANS displacement assays for FABP5 were conducted using LA and OA, and for FABP7 using LA and DHA, as representative model ligands. All fatty acids were dissolved in ethanol for these assays. Mixtures of each FABP and a 10-fold excess of ANS in buffer (10 mm potassium phosphate, 1.62 mm disodium hydrogen phosphate 2.74 mm NaCl and 40.54 mm KCl, pH 7.4) were prepared. Increasing concentrations of fatty acid were added to the mixture of FABP and ANS, and kept for 3 min, at 25°C in the dark, before measuring fluorescence. The final concentration of ethanol was kept at 0.5%. The fluorescence data were analyzed by non-linear regression analysis using GraphPad Prism software and the inhibition constant (*K*_i_) values were calculated by the tool-websites ‘The *K*_i_ calculator’ (http://sw16.im.med.umich.edu/software/calc_ki/).

### Mice

The inbred C57BL/6NCrlCrlj (B6) and C3H/HeNCrlCrlj (C3) mouse strains were obtained from Japan's Charles River Laboratories. The generation of *Fabp3*/*5*/*7*-disrupted mice is described in detail elsewhere (40,71,72). The mice used in this study were backcrossed from a mixed 129/B6 background onto B6, for at least 10 generations, and were then intercrossed to produce WT and *Fabp3*/*5*/*7*-null mice. The animals were housed in groups of four in standard cages, in a temperature and humidity-controlled room with a 12 h light/dark cycle (lights on at 08:00). The animals had free access to standard lab chow and tap water. All experiments were performed between 10:00 and 17:00. The experimental procedures were approved by the RIKEN Animal Ethics Committee.

### Behavioral analyses

Detailed procedures for behavioral phenotyping are provided as Supplementary Material.

## SUPPLEMENTARY MATERIAL

Supplementary Material is available at *HMG* online.

## FUNDING

This study was supported in part by Grants-in-Aid for Scientific Research (T.Y.) and by Grant-in-Aid for Scientific Research on Innovative Areas (T.Y.), from the Japan Society for the Promotion of Science (JSPS), Japan. In addition, this study was supported by RIKEN Brain Science Institute Funds (T.Y.), and a part of this study is the result of the ‘Development of biomarker candidates for social behavior’ (T.Y.) and ‘Integrated research on neuropsychiatric disorders’ projects (N.M.), carried out under the Strategic Research Program for Brain Sciences by the Ministry of Education, Culture, Sports, Science and Technology of Japan. Funding to pay the Open Access publication charges for this article was provided by RIKEN BSI funds.

## Supplementary Material

Supplementary Data
