# Functional characterization of *FABP3*, *5* and *7* gene variants identified in schizophrenia and autism spectrum disorder and mouse behavioral studies

**DOI:** 10.1093/hmg/ddv011

**Published:** 2015-02-05

**Authors:** Chie Shimamoto, Tetsuo Ohnishi, Motoko Maekawa, Akiko Watanabe, Hisako Ohba, Ryoichi Arai, Yoshimi Iwayama, Yasuko Hisano, Tomoko Toyota, Manabu Toyoshima, Katsuaki Suzuki, Yukihiko Shirayama, Kazuhiko Nakamura, Norio Mori, Yuji Owada, Tetsuyuki Kobayashi, Takeo Yoshikawa

*Human Molecular Genetics*, 2014 23:24; pp. 6495–6511; doi: 10.1093/hmg/ddu369

In this article, there was a mistake on p. 6506. In the MATERIALS AND METHODS, line 5 of the Human postmortem brains section: “the Maryland Psychiatric Research Center, Baltimore, MD, USA” should be corrected to: “University of Maryland Brain and Tissue Bank, a Brain and Tissue Repository of the NIH NeuroBioBank, Baltimore, MD, USA”.

The authors sincerely apologise for this error.

