# Supplementary material for: Functional characterization of FABP3, 5 and 7 gene variants identified in schizophrenia and autism spectrum disorder and mouse behavioral studies
Source: Hum Mol Genet. 2014 Jul 15;23(24):6495–511. doi: 10.1093/hmg/ddu369 (PMC4240203; doi:10.1093/hmg/ddu369)
Supplement: Supplementary Data [file supp_23_24_6495__index.html]

Functional characterization of FABP3, 5 and 7 gene variants identified in schizophrenia and autism spectrum disorder and mouse behavioral studies — Functional characterization of FABP3, 5 and 7 gene variants identified in schizophrenia and autism spectrum disorder and mouse behavioral studies — Supplementary Data 

# Functional characterization of *FABP3*, *5* and *7* gene variants identified in schizophrenia and autism spectrum disorder and mouse behavioral studies

## Supplementary Data

Supplementary Data

**Files in this Data Supplement:**

- Supplementary Figures - pdf file
- Supplementary Data - Docx file
