# Supplementary material for: Functional characterization of FABP3, 5 and 7 gene variants identified in schizophrenia and autism spectrum disorder and mouse behavioral studies
Source: Hum Mol Genet. 2014 Jul 15;23(24):6495–511. doi: 10.1093/hmg/ddu369 (PMC4240203; doi:10.1093/hmg/ddu369)

Supplementary Figure 1

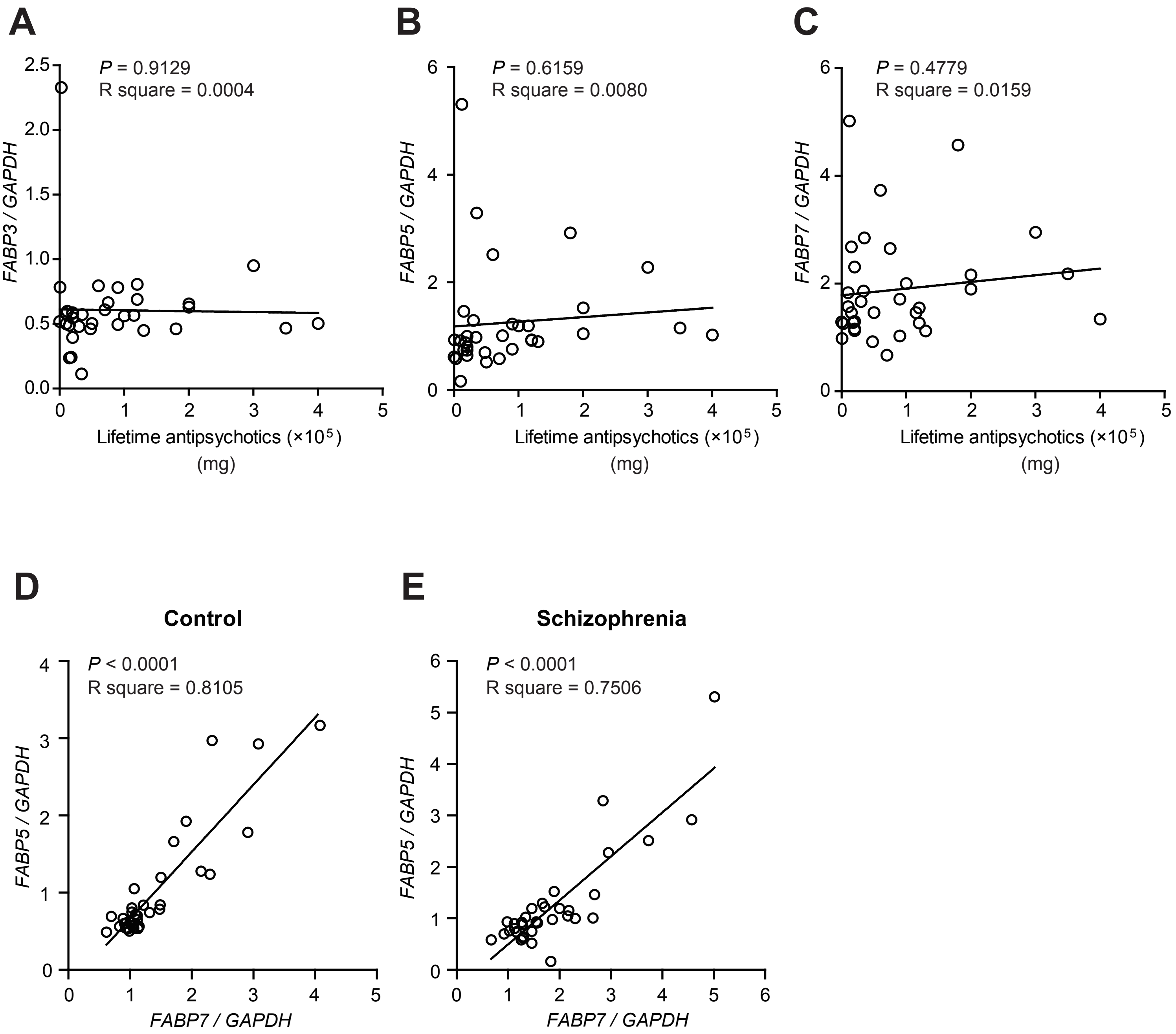

Supplementary Figure 2

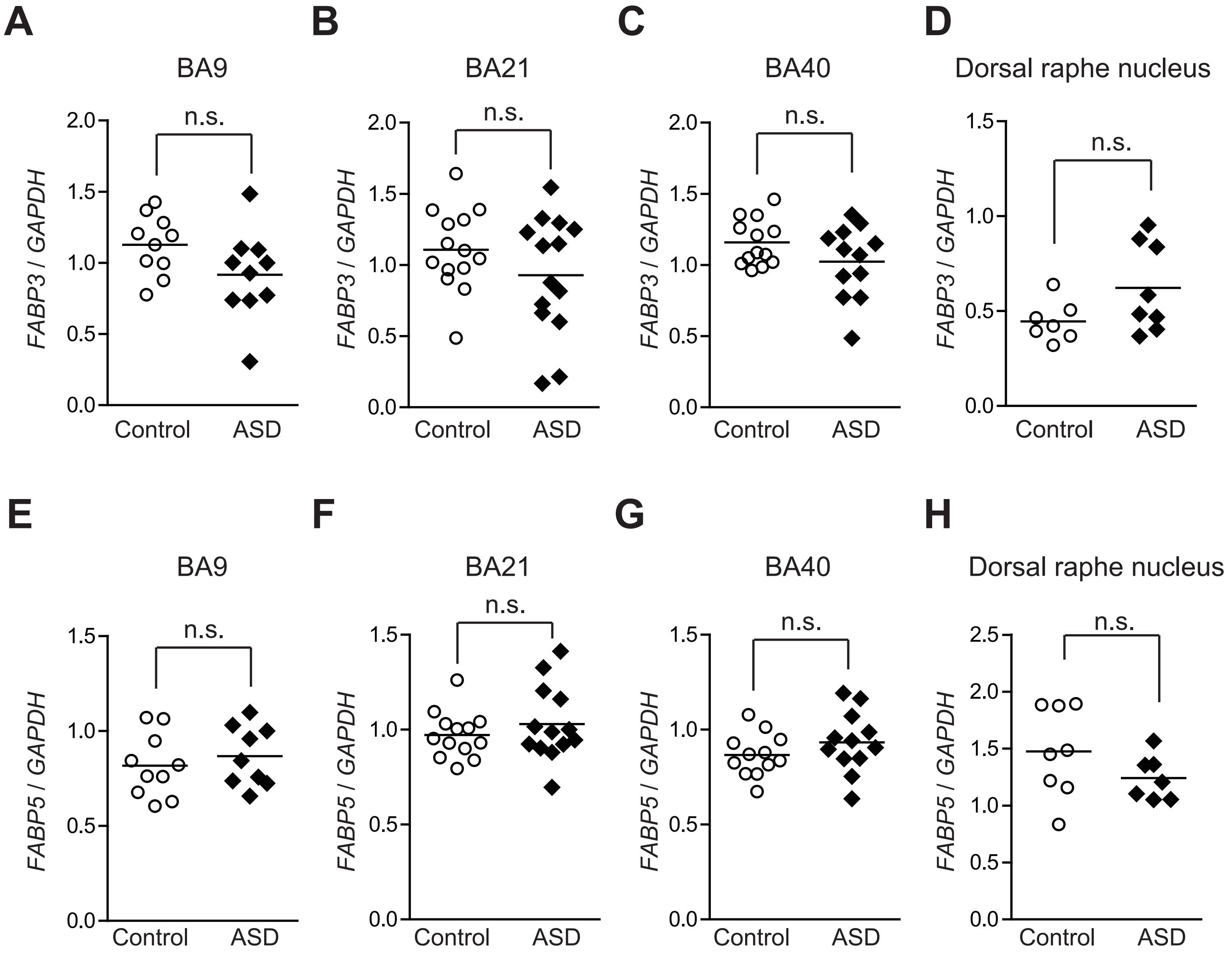

Supplementary Figure 3

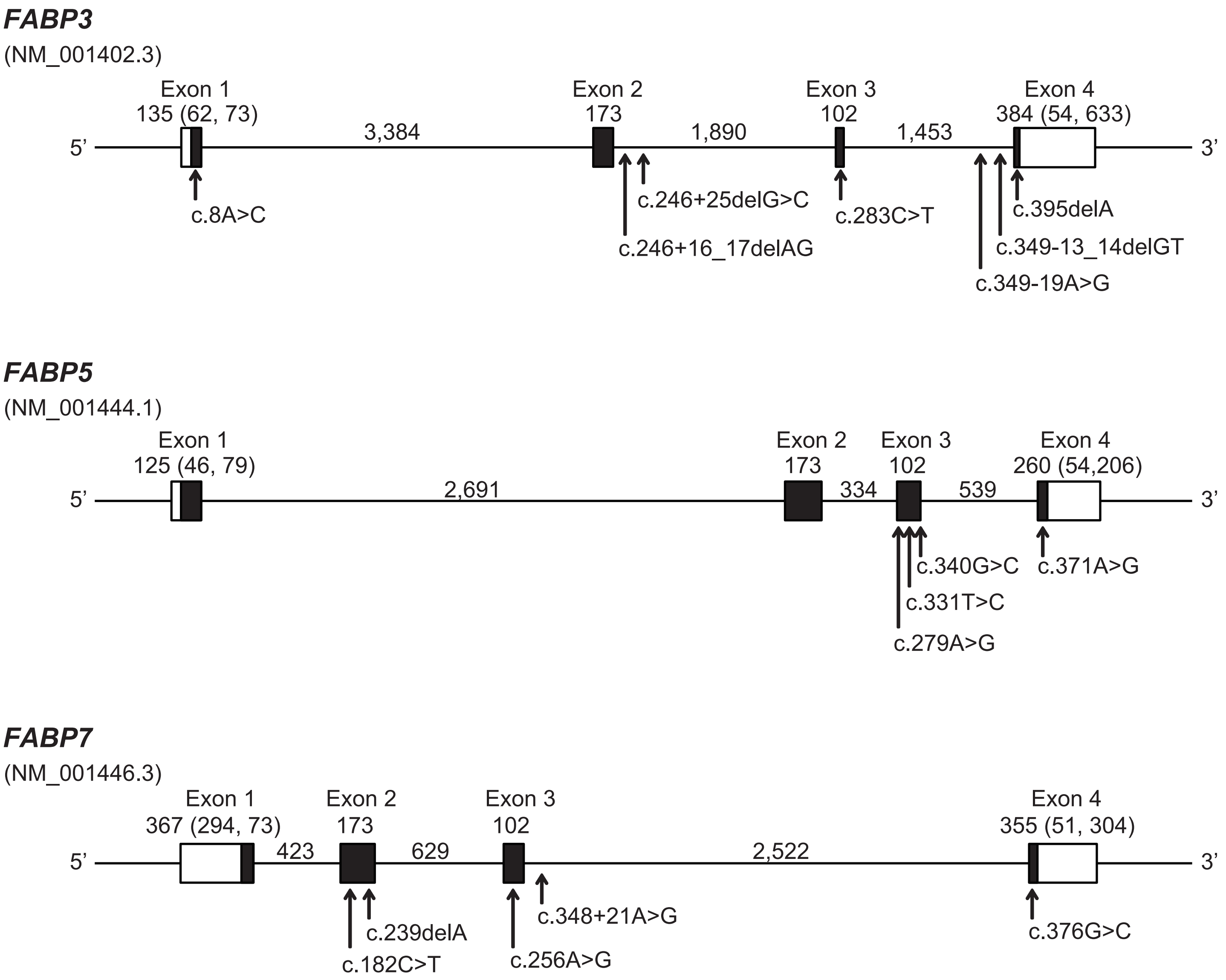

Supplementary Figure 4

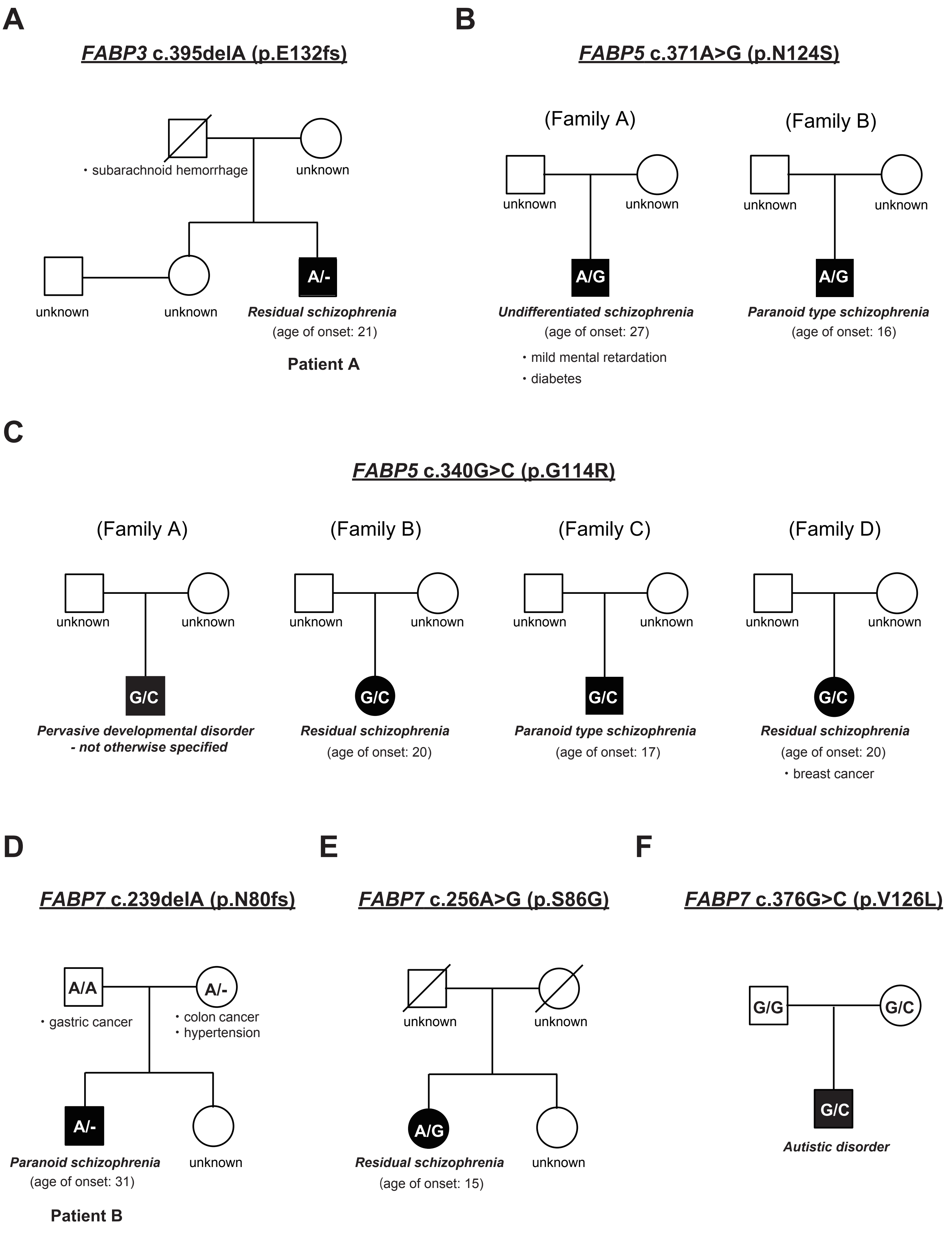

Supplementary Figure 5

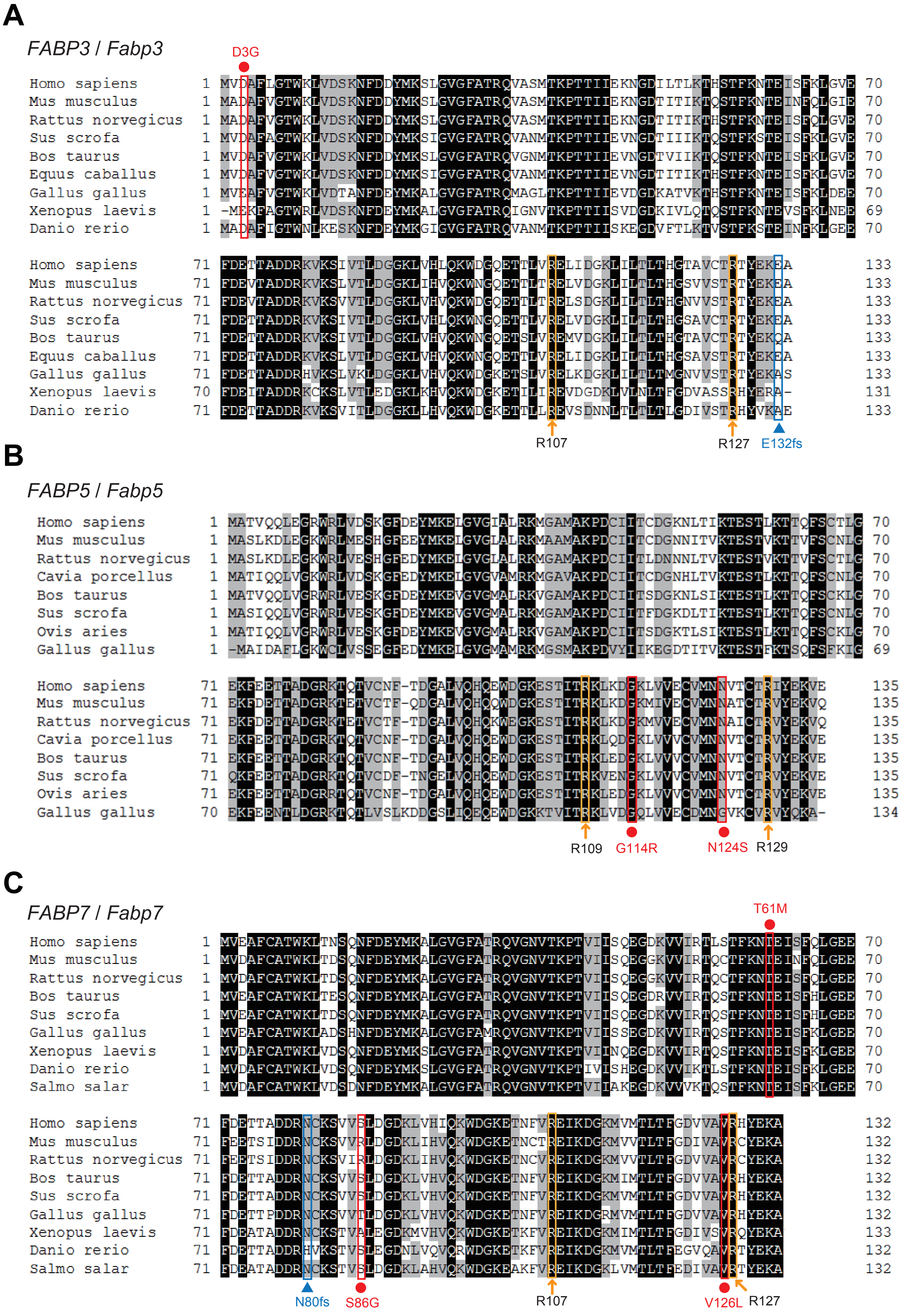

Supplementary Figure 6

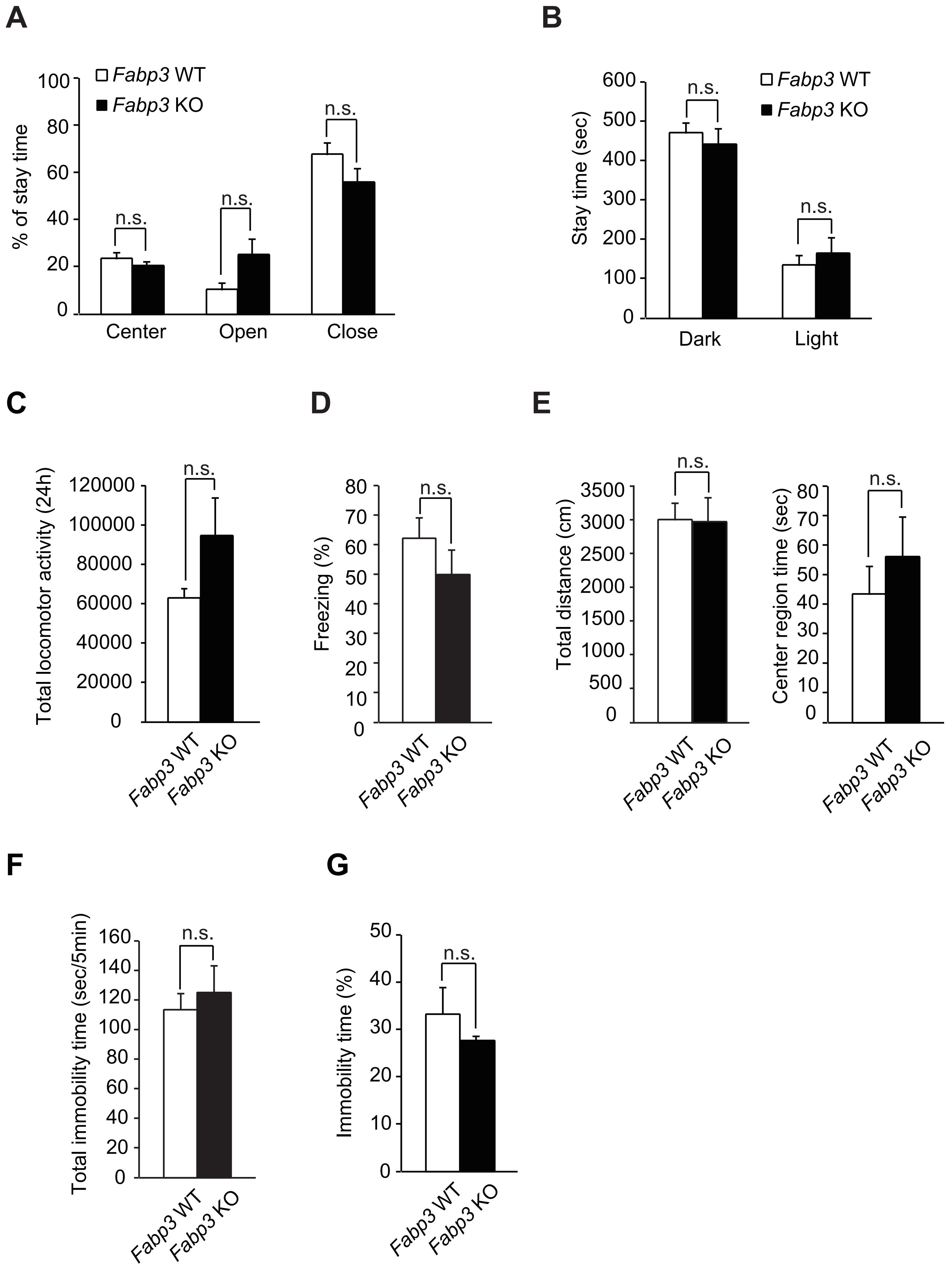

Supplementary Figure 7

A

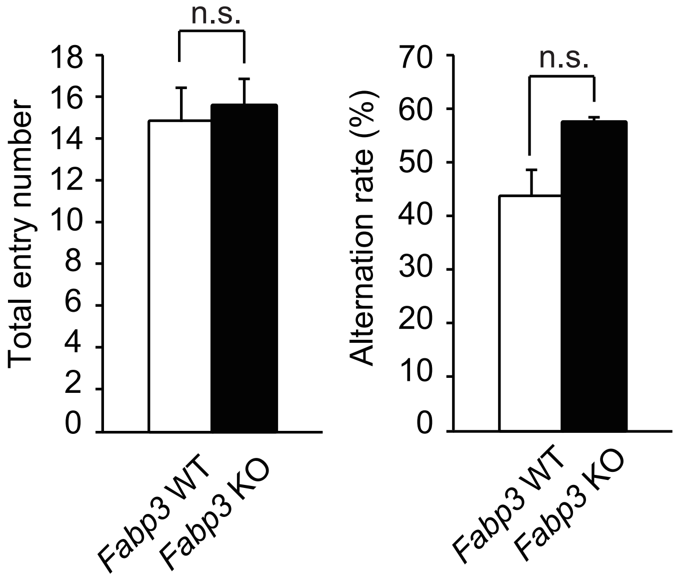

B

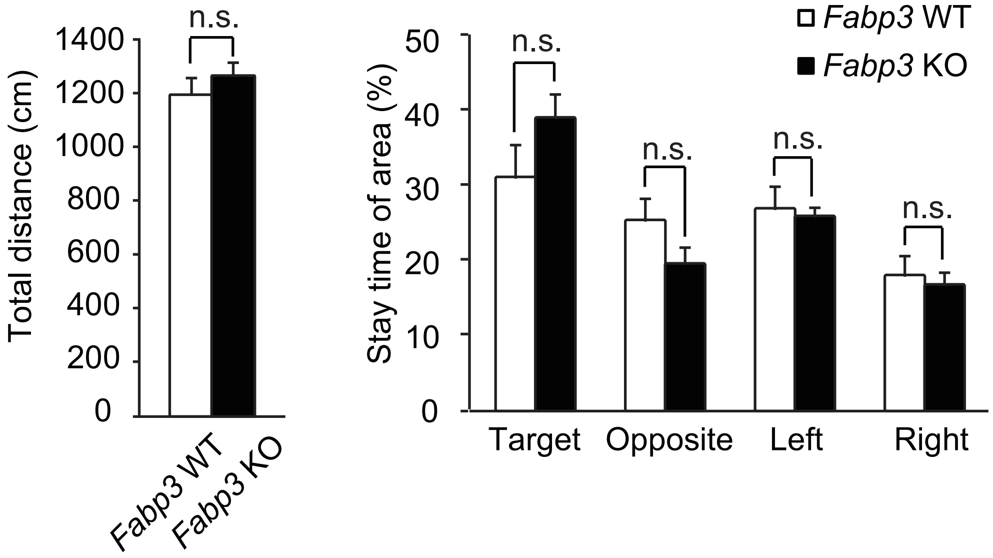

C

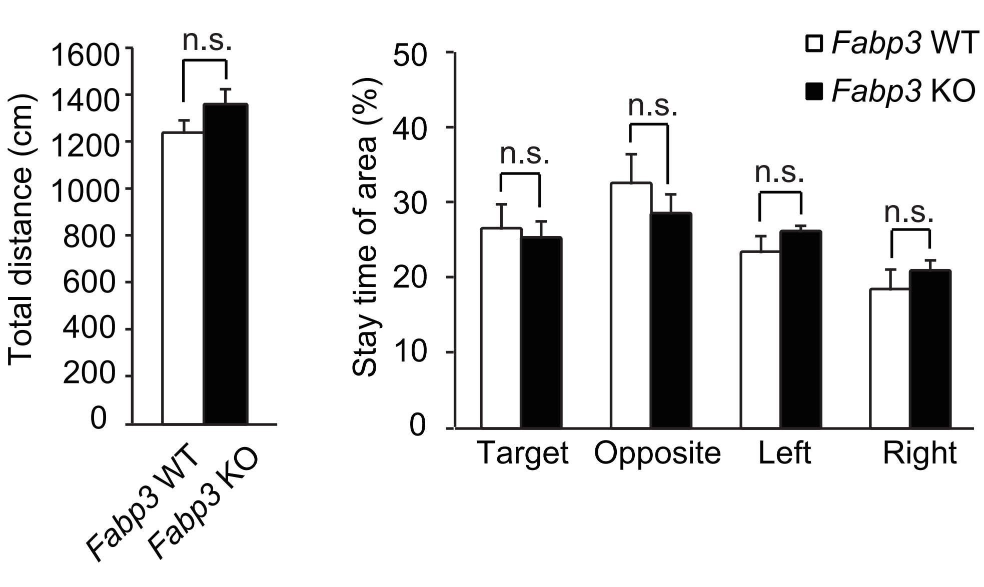

Supplementary Figure 8

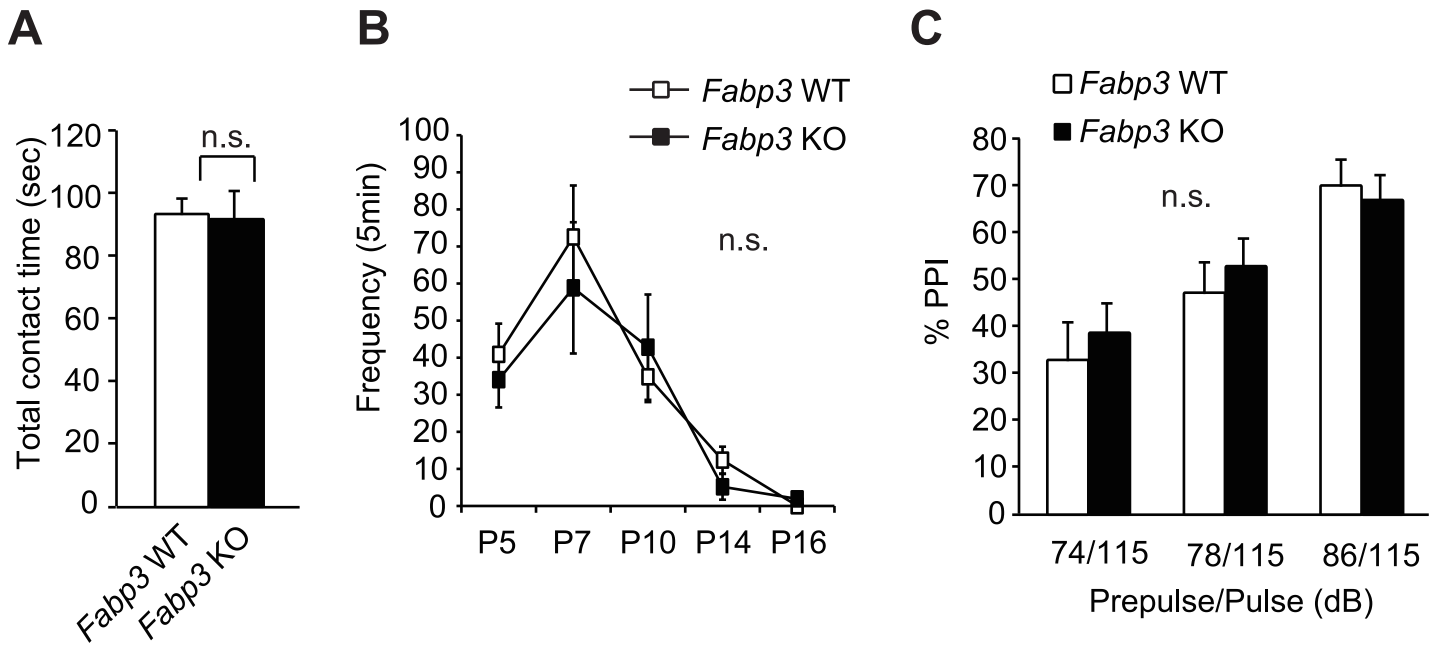

Supplementary Figure 9

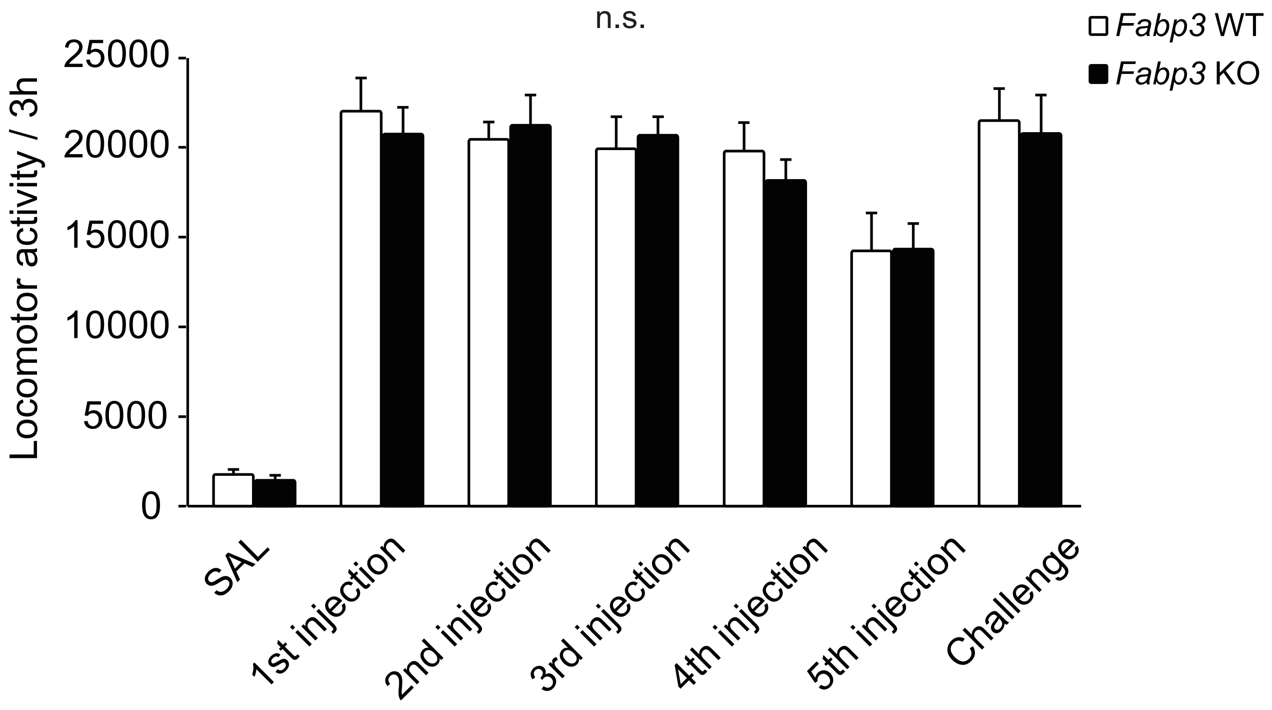

Supplementary Figure 10

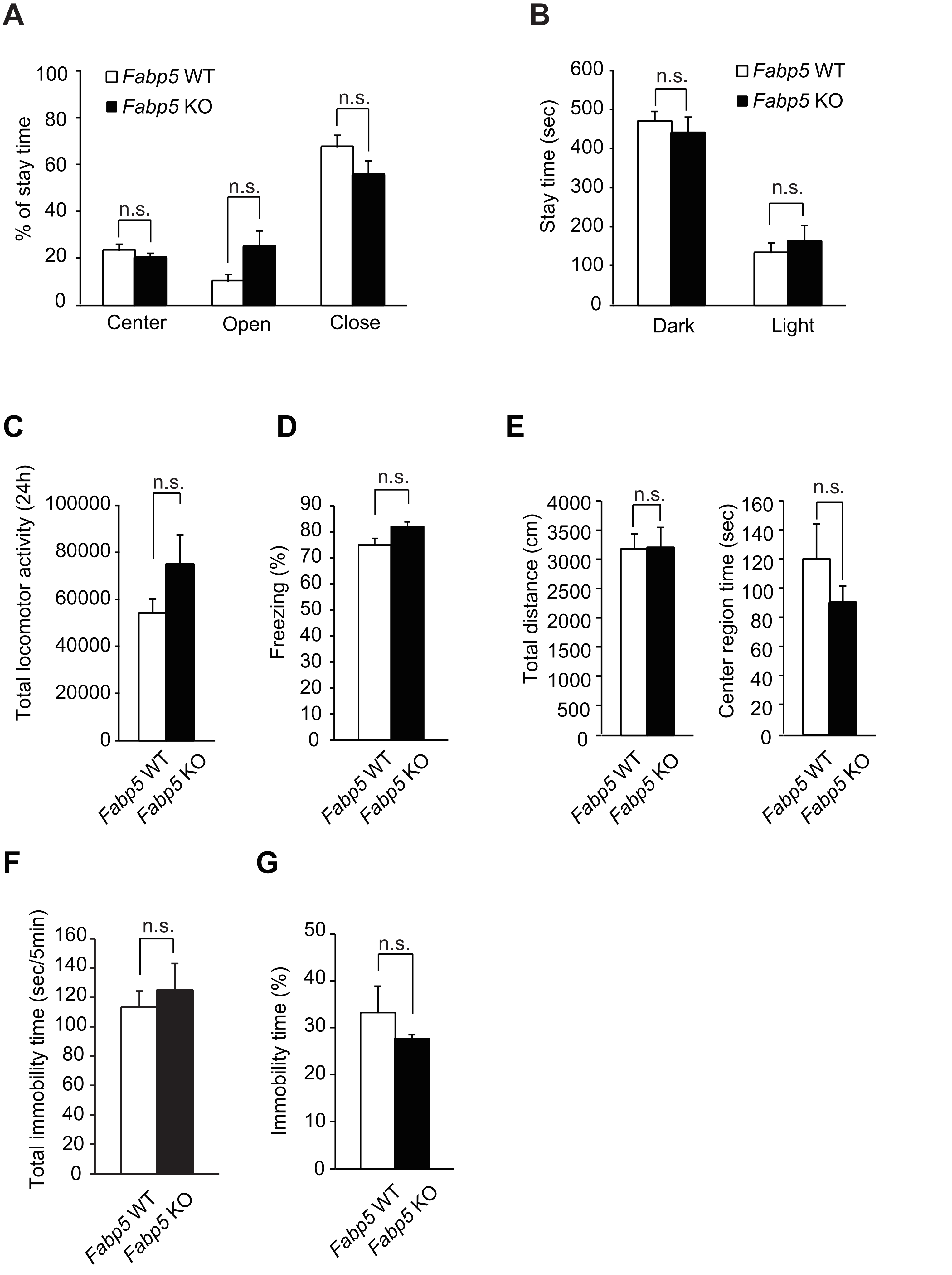

Supplementary Figure 11

A

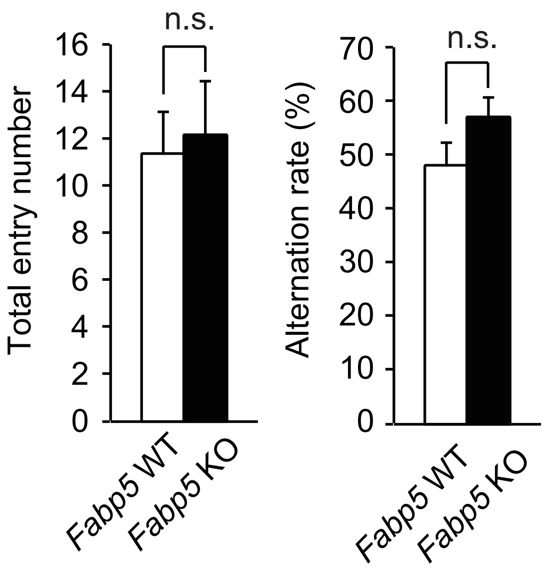

B

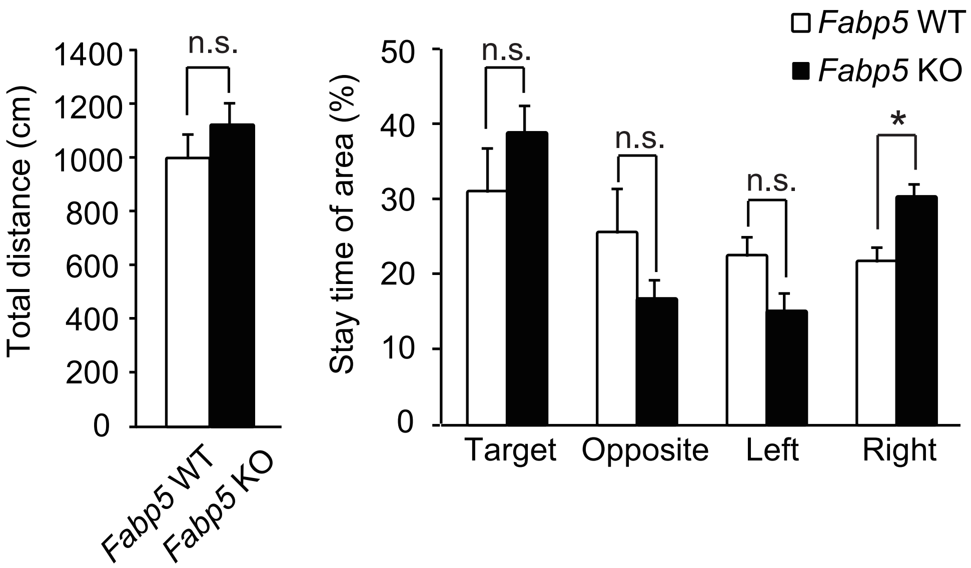

C

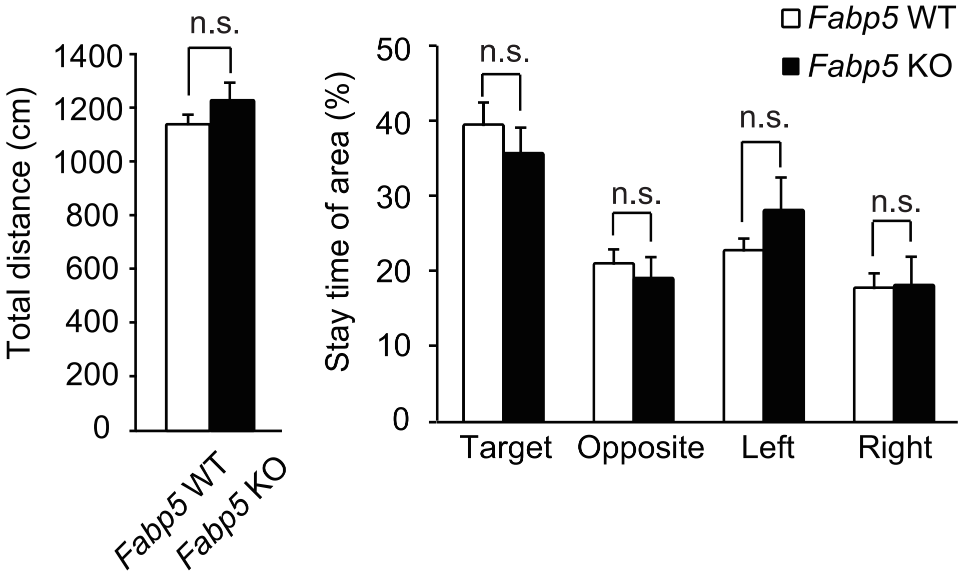

Supplementary Figure 12

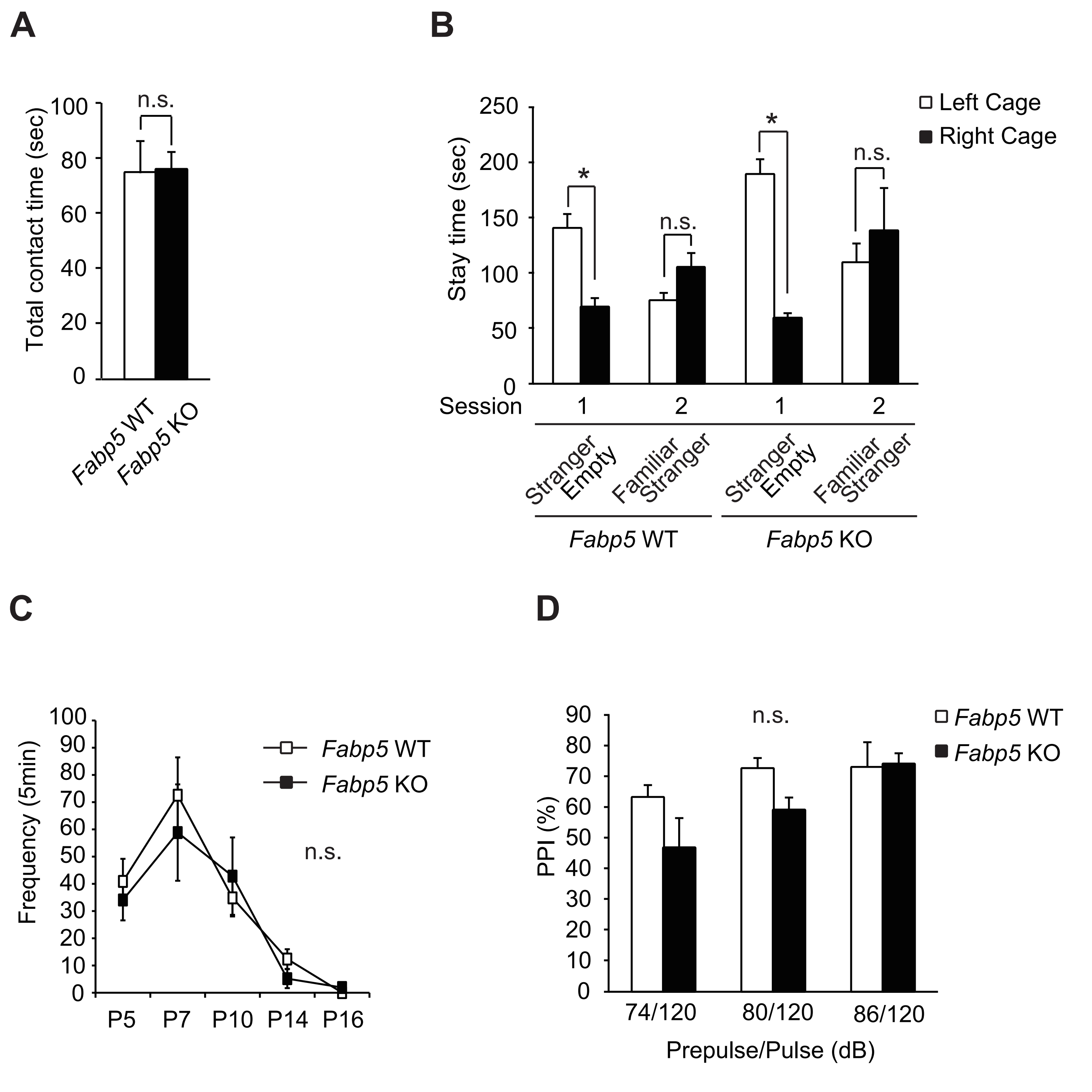

Supplementary Figure 13

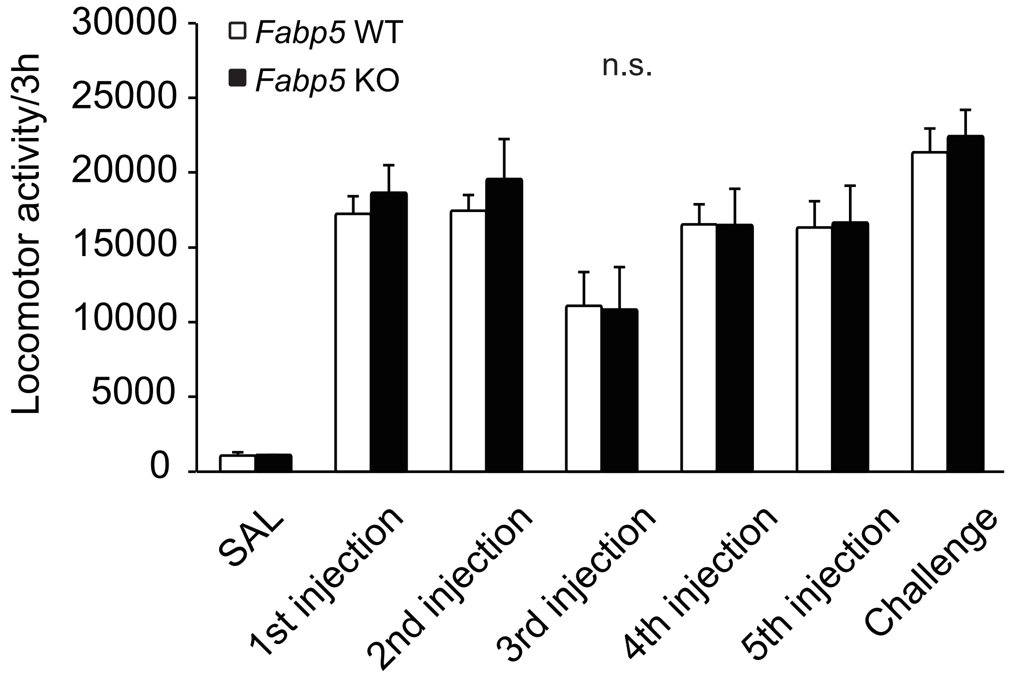

Supplementary Figure 14

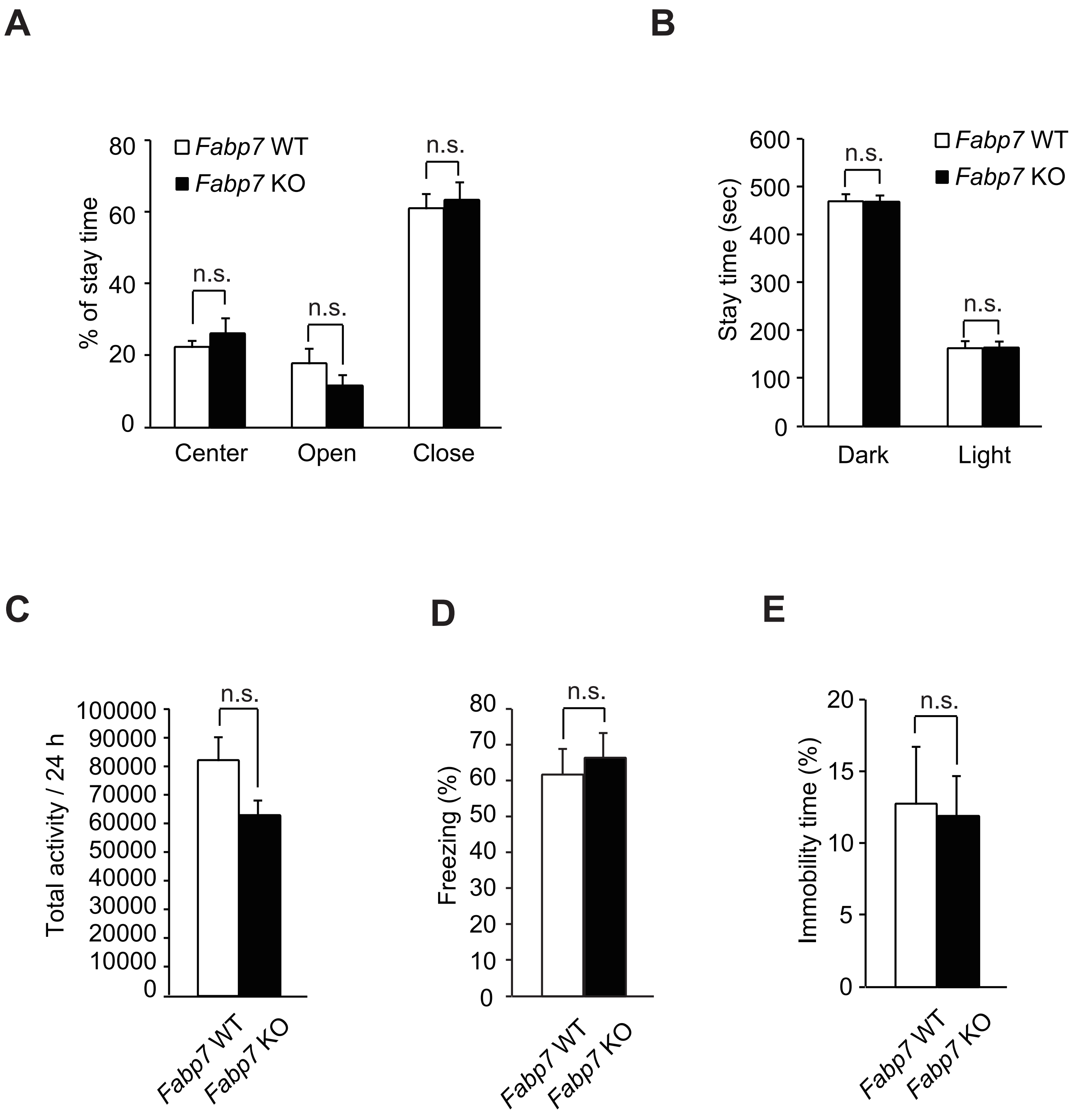

Supplementary Figure 15

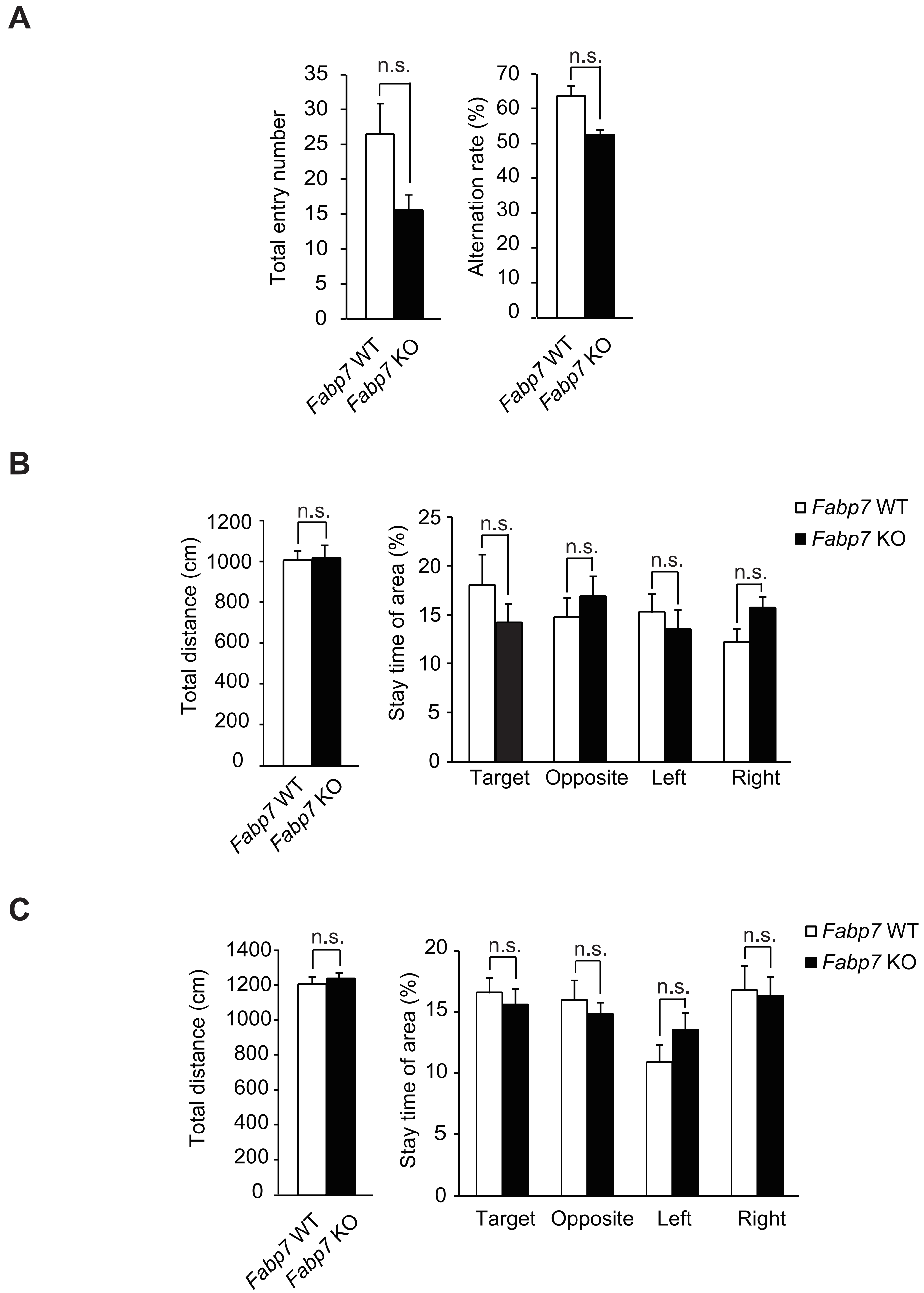

Supplementary Figure 16

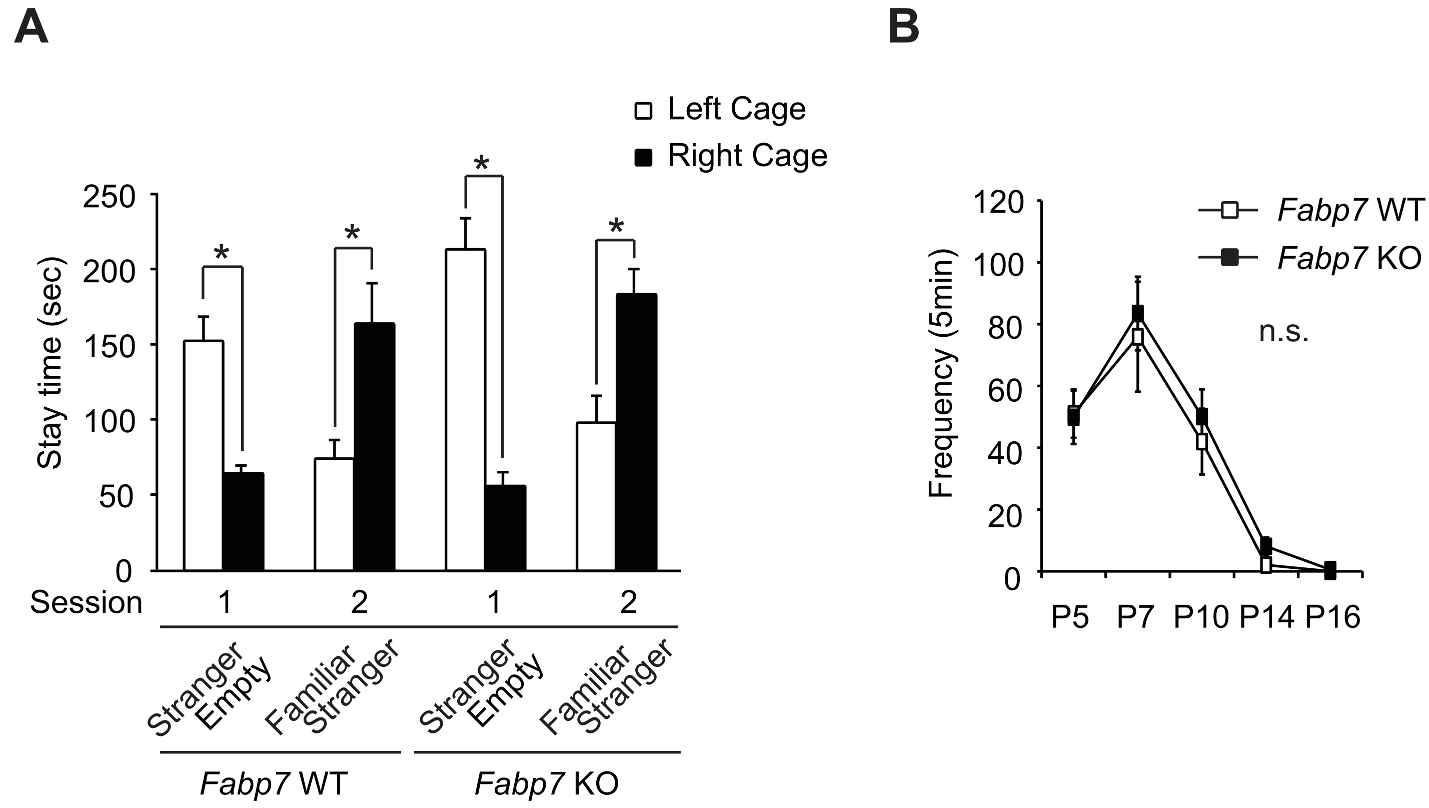

Supplementary Figure 17

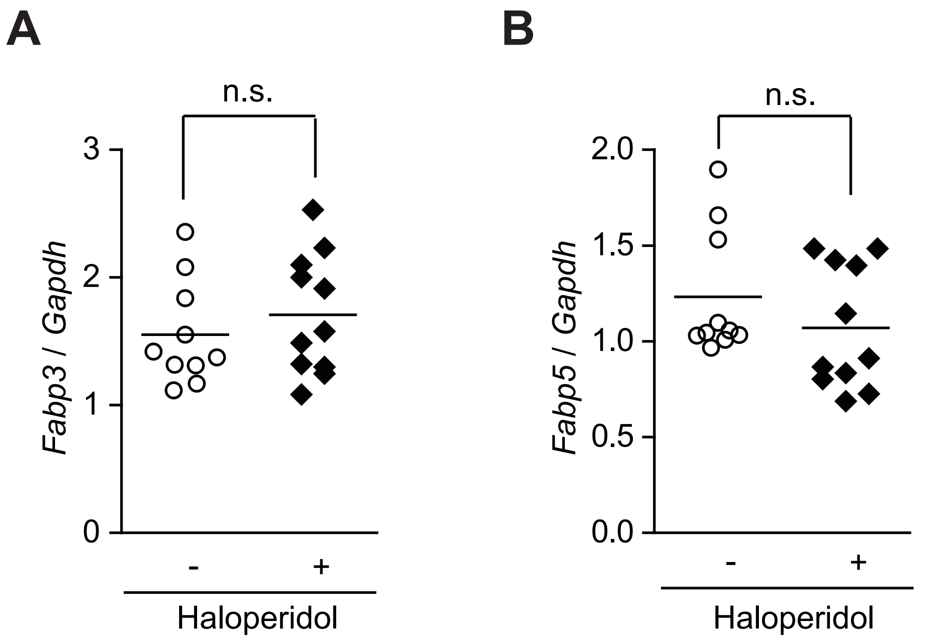

Supplement: Supplementary Data [file supp_ddu369_ddu369supp_figs.pdf]
