# Supplementary material for: Functional characterization of FABP3, 5 and 7 gene variants identified in schizophrenia and autism spectrum disorder and mouse behavioral studies
Source: Hum Mol Genet. 2014 Jul 15;23(24):6495–511. doi: 10.1093/hmg/ddu369 (PMC4240203; doi:10.1093/hmg/ddu369)
Supplement: Supplementary Data [file supp_ddu369_ddu369supp.docx]

**SUPPLEMENTARY MATERIAL**

**SUPPLEMENTARY EXPERIMENTAL PROCEDURES**

**Behavioral phenotyping**

***Open field, home cage activity and prepulse inhibition (PPI) tests***

The open field test was conducted according to a method previously reported ([1](#_ENREF_1)), using the automatic monitoring system, TimeOFC4 (O’Hara, Tokyo, Japan). The home cage activity test was conducted using a method previously reported ([1](#_ENREF_1)). PPI testing was based on a procedure previously described ([2](#_ENREF_2)), with the exception that a startle reflex measurement system (O’Hara) was used.

***Forced swim and tail suspension tests***

Forced swim test was conducted using a method previously reported ([3](#_ENREF_3)) with minor modifications. Mice were forced to swim for 5 min in a transparent glass cylinder, containing water to a depth of 10 cm. The tail suspension test was conducted using a previously reported method ([3](#_ENREF_3)) with minor modifications. In the current study, data for the first min of a 10 min session were excluded and the immobility times in the remaining 9 min were summed.

***Elevated plus maze* *and* *light and dark box tests***

Elevated plus-maze test was conducted as previously reported ([1](#_ENREF_1)), using Image Time EP4 (O’Hara). The light-dark box test was performed as described elsewhere ([1](#_ENREF_1)) with minor modifications, Briefly, the Time LD4 system (O’Hara) was used to record latency before entering a light compartment (illuminated at 200 lux) and to calculate the distance traveled within each compartment.

***Fear conditioning test***

A mouse was placed in a test chamber (15 x 15 x 15 cm), consisting of white plastic walls with a stainless steel floor. A CCD camera was attached to the ceiling of the chamber and connected to a video monitor and computer. An auditory cue at 65 dB, 10 kHz was supplied from a loudspeaker (CS: conditioned stimulus). The conditioning trial consisted of a 2 min exploration period followed by two CS-US (unconditioned stimulus) pairings, separated by 1 min. An US (foot shock: 0.5 mA, 2 sec) was administered at the end of the 30 sec CS period. Contextual testing was conducted one day after conditioning, in the same chamber for 3 min. The rate of freezing response (immobility excluding respiration and heartbeat) in mice was measured as an index of fear memory. Data were collected and analyzed using Image J FZ2 (O’Hara) and Time FZ2 (O’Hara).

***Resident-intruder test***

Mice were individually housed in a home cage (29 x 18 x 12 cm) for 2 days before trials. We used 10–15 week old male mice who had not previously shown aggressive behavior as intruders. In the first trial (5 min duration), an intruder mouse was introduced into a resident's home cage under bright light conditions (70 lux). The duration of social interaction (close following, inspection, anogenital sniffing and other social body contacts excluding aggressive behavior), aggression (attacking/biting and tail rattling) and escape behavior were analyzed visually.

***Three-chamber and Y maze tests***

Three-chambered social interaction test was based on Crawley’s procedure ([4](#_ENREF_4)), using an apparatus consisting of a rectangular, three-chambered box and a lid with a CCD camera (Time CSI; O’Hara). We examined short-term memory using the Y-maze test as described ([5](#_ENREF_5))

***Morris water maze***

The hidden platform, probe test, and reversal probe test components of the Morris water task were conducted in a circular pool, 100 cm in diameter (Time MGM; O’Hara). Water maintained at 22.5-23.5℃ was rendered opaque by the addition of nontoxic white paint. Video tracking was conducted with a camera focused on the full diameter of a pool. Each training trial was performed by placing the mouse into the quadrant that was either right of, left of, or opposite to a target containing a platform. A different order of start positions was applied every day, however, an identical order of start positions was used for all mice. Training trials were of a 60 sec maximum duration. If a mouse failed to reach the platform within 60 sec, it was then guided to the platform. Four trials per day were conducted for 5 days for acquisition of the hidden platform task, with the original platform location and 5 days with a new platform location (reversal probe test), rotated by 180-degrees from the original platform location. 24h after the end of the hidden platform training, a probe test and a reversal probe test were conducted for 1 min to confirm that this spatial task was acquired, based on navigation by distal environmental room cues. Distance traveled to the platform was automatically recorded.

***Ultrasonic vocalization (USV)***

USV was recorded in a soundproof box with a condenser ultrasound microphone (CM16/CMPA, Avisoft Bioacoustics, Berlin, Germany). The microphone was connected to an amplifier/digitizer (UltraSoundGate116H, Avisoft Bioacoustics, Berlin, Germany). The recorded files were transferred to the SASLab Pro Recorder Software (Avisoft Bioacoustics) for fast Fourier transform (512 FFT-length, 100% frame size, Hamming window, 50% time window overlap). The number of calls was analyzed. For the measurement of infant isolation calls, male pups were recorded at P5, P7, P10, P14 and P16. The homecage with a dam and her pups was moved to the experimental room at least 1 h before recordings. In test sessions, the dam was removed and the pups were maintained at room temperature in a homecage. Each pup was put into a plastic beaker (9 cm diameter, 12 cm height) and placed in a soundproof box. USV was recorded for 5 min at a sampling frequency of 300 kHz.

***Effects of MK-801 administration on locomotor activity***

Dizocilpine maleate (MK-801) (Tocris Bioscience, Bristol, United Kingdom) was dissolved in 0.9% saline solution. Mice were injected subcutaneously with 0.3 mg/kg of MK-801 once daily for 5 days. At 7 days after the final MK-801 administration, mice received a challenge injection. Locomotor activity was measured using an infrared sensor (Supermex; Muromachi Kikai, Tokyo, Japan) after each challenge dosing.

**Effects of haloperidol administration on transcript levels of *Fabp3* and *Fabp5***

The typical antipsychotic haloperidol (Dainippon Pharmaceutical, Osaka, Japan) was administered to C3H/HeNCrlCrlj (C3) male mice in drinking water at a concentration of 0.1 mg/day for 3 weeks. Control C3 male mice were given drinking water without haloperidol. On day 21, the mice were decapitated and the frontal cortex was quickly dissected out. Transcript levels of *Fabp3* and *5* were examined as described above.

**LEGENDS TO SUPPLEMENTARY FIGURES**

**Supplementary Figure 1. Correlation analyses between expression levels of brain-expressed *FABP*s and lifetime antipsychotics, and between expression levels of *FABP5* and those of *FABP7* in postmortem brains**

No correlations are seen between *FABP3* (**A**), *FABP5* (**B**) and *FABP7* (**C**) expression levels and lifetime antipsychotics, in schizophrenic postmortem brains. The dose of lifetime antipsychotics is shown as fluphenazine equivalent. Correlations between expression levels of *FABP5* (examined in Fig. 1B) and *FABP7* [examined in Watanabe *et al. (*[*2*](#_ENREF_2)*)*] in postmortem brains (BA 46) from controls (**D**) and schizophrenic patients (**E**) were tested. *GAPDH* was used as an internal control.

**Supplementary Figure 2. Transcript levels of *FABP3* and *FABP5* in postmortem brains from autistic patients and controls**

(**A**-**D**) Transcript levels of *FABP3* in postmortem brains from autistic patients and controls. We examined four regions of postmortem brains; BA9: control (n = 10) and ASD samples (n = 10) (A), BA21: control (n = 14) and ASD samples (n = 14) (B), BA40: control (n = 13) and ASD samples (n = 13) (C) and the dorsal raphe nucleus: control (n = 7) and ASD samples (n = 8) (D). (**E**-**H**) Transcript levels of *FABP5* in postmortem brains from autistic patients and controls; BA9: control (n = 10) and ASD samples (n = 9) (E), BA21: control (n = 13) and ASD samples (n = 13) (F), BA40: control (n = 12) and ASD samples (n = 12) (G) and the dorsal raphe nucleus: control (n = 8) and ASD samples (n = 7) (H). *GAPDH* was used as an internal control. The gene expression levels were evaluated as in Fig. 1.

**Supplementary Figure 3. Genomic structure and polymorphic sites in *FABP* genes**

Exons are denoted as boxes, with coding regions in black and untranslated regions in white. The sizes of each exon and intron are also shown.

**Supplementary Figure 4. Pedigrees of patients with mutations in brain-expressed *FABP*s**

(**A**) Family structure of a patient carrying *FABP3* c.395delA (p.E132fs). More detailed information is described in the supplementary note (patient A). (**B**) Two family pedigrees of patients carrying *FABP5* c.371A>G (p.N124S). (**C**) Four family pedigrees of patients harboring *FABP5* c.340G>C (p.G114R). (**D**) Family pedigree of a patient with *FABP7* c.239delA (p.N80fs). More detailed information is described in the supplementary note (patient B). The mutant allele was transmitted from the patient’s mother. (**E**) Family pedigree of a patient harboring *FABP7* c.256A>G (p.S86G). (**F**) Family pedigree of a patient harboring *FABP7* c.376G>C (p.V126L). The mutant allele was transmitted from the patient’s mother. Squares indicate males; circles, females; diagonal slashes, deceased individuals; black filled symbols, patients with a psychiatric disorder. Genotype and clinical information are also shown.

**Supplementary Figure 5. Amino acid sequence alignments of *FABP*s**

Amino acid sequences of fatty acid binding protein 3 (**A**), 5 (**B**) and 7(**C**) from multiple vertebrate species are shown. The positions of the missense mutations and frameshift starting points are denoted as red circles and blue triangles, respectively.

**Supplementary Figure 6. Anxiety related behavior and spontaneous locomotor activity in *Fabp3* KO mice**

(**A**) Elevated plus maze test. The graph shows the percentage of stay time in the center of arms, and in open and closed arms. (**B**) Light and dark box test. The graph shows the stay time (sec) in dark and light chambers. (**C**) Home cage activity test. The graph shows total locomotor activity in 24 h. (**D**) Fear conditioning test. The graph shows the percentage of freezing time in the contextual test. (**E**) Open field test. Left graph shows total distances (cm) in 10 min and the right graph, the time spent in the center area. (**F**) Forced-swim test. The graph shows total immobility time (sec) over a 5 min period. (**G**) Tail suspension test. The graph shows the percentage of immobility. Values are mean ± SEM (n = 10-14/group). Data were evaluated using the two-tailed Mann-Whitney *U* test.

**Supplementary Figure 7. Reference memory ability in *Fabp3* KO mice**

(**A**) Y-maze test. Left and right graphs show total arm entry number and alternation rates (%), respectively. (**B, C**) Morris water maze. The results of the reverse probe test and probe test are shown in (B) and (C), respectively. The maze is divided into 4 equal quadrants as indicated by each circle: target, opposite, left, and right. Values are mean ± SEM (n = 10-14/group). Data were evaluated using two-tailed Mann-Whitney *U* test.

**Supplementary Figure 8. Schizophrenia and ASD related behavior in *Fabp3* KO mice**

(**A**) Resident intruder test. The graph shows mean ± SEM of total contact time (sec) of residents (WT and KO mice) towards intruders. WT; n = 13, KO; n = 11. Data were evaluated using two-tailed Mann-Whitney *U* test. (**B**) Ultrasonic vocalization test. The graph shows mean ± SEM of the frequency of total vocal calls between P5 and P16. WT; n = 18-19, KO; n = 12-14. (**C**) Prepulse inhibition test. The graph shows mean ± SEM of PPIs (%). WT; n = 13, KO; n = 11. Two-way repeated measures ANOVA detected no genotype effect. (B, C).

**Supplementary Figure 9. Locomotor activity after injections of MK-801 in *Fabp3* KO mice**

The graph shows locomotor activity measured over 3h after a single injection of saline, five single daily injections of MK-801 (0.3 mg/kg) and a challenge injection (0.3 mg/kg) after the five repeated treatments. Values are mean ± SEM (n = 12/group). Two-way repeated measures ANOVA detected no genotype effect.

**Supplementary Figure 10. Behavioral analysis of anxiety related behavior and spontaneous locomotor activity in *Fabp5* KO mice**

(**A**) Elevated plus maze test. The graph shows the percentage of stay time in the center of the arms, and in the open and closed arms. (**B**) Light and dark box test. The graph shows the stay time (sec) in the dark and light chamber. (**C**) Home cage activity test. The graph shows total locomotor activity in 24 h. (**D**) Fear conditioning test. The graph shows the freezing time (%) in the contextual test. (**E**) Open field test. The left graph shows total distances (cm) of movement over a 10 min period, and right graph the time spent in a center area. (**F**) Forced swim test. The graph shows total immobility time (sec) over a 5 min period. (**G**) Tail suspension test. The graph shows the percentage of immobility time. Values are mean ± SEM (n = 8-13/group). Data were evaluated using the two-tailed Mann-Whitney *U* test.

**Supplementary Figure 11. Reference memory ability in *Fabp5* KO mice**

(**A**) Y-maze test. Left graph shows total arm entry number and the right graph, the alternation rates (%). (**B, C**) Morris water maze test. Results of the probe test are shown in (B) and results of the reverse probe test are shown in (C). The maze is divided into 4 equal quadrants as indicated by each circle: target, opposite, left, and right. Values are mean ± SEM (n = 8-11/group). Data were evaluated using two-tailed Mann-Whitney *U* test. **P* < 0.05

**Supplementary Figure 12. Social interaction and schizophrenia and ASD related behavior in *Fabp5* KO mice**

(**A**) Resident intruder test. The graph shows mean ± SEM of total contact time (sec) of residents (WT and KO mice) towards intruders. WT; n=10, KO; n=9. (**B**) Three-chamber test. Session 1: social affiliation and sociability. Mean stay time ± SEM in a chamber with a stranger (stranger) and in an opposite chamber (empty). Session 2: social memory and novelty seeking. Mean stay time ± SEM in the chamber with the mouse used in session I (familiar) and in the opposite chamber with a new unfamiliar mouse (stranger). WT; n = 13, KO; n = 9. (**C**) Ultrasonic vocalization test. The graph shows mean ± SEM of the frequency of total vocal calls between P5 and P16. WT; n = 8-15, KO; n = 5-9. (**D**) Prepulse inhibition test. The graph shows mean ± SEM of PPI (%). WT; n = 6, KO; n = 4. Data were evaluated using two-tailed Mann-Whitney *U* test (A, B) **P* < 0.05. Two-way repeated measures ANOVA detected no genotype effect (C, D).

**Supplementary Figure 13. Locomotor activity after injections of MK-801 in *Fabp5* KO mice**

The graph shows locomotor activity measured over 3 h, after five single injections of MK-801 (0.3 mg/kg) and a challenge injection of MK-801 (0.3 mg/kg), following five repeated treatments. Values are mean ± SEM. WT; n = 8-11, KO; n = 6-9. Two-way repeated measures ANOVA detected no genotype effect.

**Supplementary Figure 14. Anxiety related behavior and spontaneous locomotor activity in *Fabp7* KO mice**

(**A**) Elevated plus maze test. The graph shows the percentage of stay time in the center of arms, and in open and closed arms. (**B**) Light and dark box test. The graph shows the stay time (sec) in the dark and light chambers. (**C**) Home cage activity test. The graph shows total locomotor activity in 24 h. (**D**) Fear conditioning test. The graph shows the freezing time (%) in the contextual test. (**E**) Tail suspension test. The graph shows the percentage immobility time. Values are mean ± SEM (n = 16-20/group). Data were evaluated using two-tailed Mann-Whitney *U* test.

**Supplementary Figure 15. Reference memory ability in *Fabp7* KO mice**

(**A**) Y-maze test. The left graph shows total arm entry number and the right graph, the alternation rates (%). (**B, C**) Morris water maze test. The results of probe tests are shown in (B) and the results of reverse probe tests are shown in (C). The maze is divided into 4 equal quadrants as indicated by each circle: target, opposite, left, and right. Values are mean ± SEM (n = 8-11/group). Data were evaluated using two-tailed Mann-Whitney *U* test.

**Supplementary Figure 16. Social interaction and ultrasonic vocalization in *Fabp7* KO mice.**

(**A**) Three chamber tests. Session 1: social affiliation and sociability. Mean stay time ± SEM in a chamber with a stranger (stranger) and in an opposite chamber (empty). Session 2: social memory and novelty seeking. Mean stay time ± SEM in a chamber with the mouse used in session I (familiar) and in the opposite chamber with a new unfamiliar mouse (stranger). WT; n = 13, KO; n = 9. Data were evaluated using two-tailed Mann-Whitney *U* test. **P* < 0.01. (**B**) Ultrasonic vocalization test. The graph shows mean ± SEM of the frequency of total vocal calls between P5 and P16. WT; n = 8-15, KO; n = 5-9. Two-way repeated measures ANOVA detected no genotype effect.

**Supplementary Figure 17. Transcript levels of *Fabp3* and *Fabp5* in the frontal cortex of mice treated with haloperidol**

(**A, B**) Haloperidol (0.1 mg / day) was administered to C3 male mice (n=11) in drinking water for 3 weeks. Control C3 male mice (n = 10) were given drinking water without haloperidol. On day 21, mice were decapitated, and two brain regions (frontal cortex and hippocampus) were quickly dissected out. Transcript levels of *Fabp3* (A) and *Fabp5* (B) in the frontal cortex were examined. *Gapdh* was used as an internal control. The gene expression levels were evaluated using two-tailed Mann-Whitney *U* test.

**SUPPLEMENTARY NOTE**

**Clinical descriptions of patients harboring frameshift variants in *FABP3* and *FABP7***

**Patient A [FABP3 c.395delA (p.E132fs)]**

Patient A was a Japanese male carrying a heterozygous frameshift mutation (c.395delA) in *FABP3*, who suffered from residual schizophrenia. At the study evaluation point, he was 49 year old and been treated with psychotropic drugs. He also used a lipid-lowering drug to treat hyperlipidemia. Details of his disease process, drug therapy and familial history of psychiatric illness were unknown. From blood chemical analyses, he showed a high triglyceride concentration (204–401 mg/dL, reference range 30-147 mg/dL) and a normal total cholesterol concentration (197-224 mg/dL, reference range 134-223 mg/dL).

**Patient B [FABP7 c.239delA (p.N80fs)]**

Patient B was a Japanese male carrying a heterozygous frameshift mutation (c.239delA) in *FABP7*, who suffered from paranoid type schizophrenia. At the age of 31, he began to suffer from persecutory delusions, auditory hallucinations and a disturbance of ego. At the age of 32 years, he was diagnosed with schizophrenia and prescribed medication. At the age of 36, he was admitted to hospital after a suicide attempt. On discharge, he continued to receive outpatient care. At the study evaluation point, he was 47 year old and on an antipsychotic treatment regime of 6 mg/day risperidone, 2.4 mg/day alprazolam, 3 mg/day etizolam, 10 mg/day olanzapin, 12.5 mg/day chlorpromazine hydrochloride, 30 mg/day phenobarbital, 12.5mg/day promethazine hydrochloride and 3 mg/day biperiden. He also suffered from allergic rhinitis and hyperlipidemia. There was no family history of psychiatric disease. His electroencephalogram was normal with no intracranial lesion. He had a high total cholesterol concentration (226 mg/dL, reference range 134-223 mg/dL), a high free fatty acid concentration (904 μEq/L, reference range 140-850 μEq/L) and a normal triglyceride concentration (124 mg/dL, reference range 30-147 mg/dL).

**SUPPLEMENTARY REFERENCES**

1 Ohnishi, T., Watanabe, A., Ohba, H., Iwayama, Y., Maekawa, M. and Yoshikawa, T. (2010) Behavioral analyses of transgenic mice harboring bipolar disorder candidate genes, IMPA1 and IMPA2. *Neurosci. Res.*, **67**, 86-94.

2 Watanabe, A., Toyota, T., Owada, Y., Hayashi, T., Iwayama, Y., Matsumata, M., Ishitsuka, Y., Nakaya, A., Maekawa, M., Ohnishi, T. *et al.* (2007) Fabp7 maps to a quantitative trait locus for a schizophrenia endophenotype. *PLoS Biol.*, **5**, e297.

3 Yoshikawa, T., Watanabe, A., Ishitsuka, Y., Nakaya, A. and Nakatani, N. (2002) Identification of multiple genetic loci linked to the propensity for "behavioral despair" in mice. *Genome Res.*, **12**, 357-366.

4 Crawley, J.N. (2004) Designing mouse behavioral tasks relevant to autistic-like behaviors. *Ment Retard Dev Disabil Res Rev*, **10**, 248-258.

5 Takeuchi, H., Iba, M., Inoue, H., Higuchi, M., Takao, K., Tsukita, K., Karatsu, Y., Iwamoto, Y., Miyakawa, T., Suhara, T. *et al.* (2011) P301S mutant human tau transgenic mice manifest early symptoms of human tauopathies with dementia and altered sensorimotor gating. *PLoS One*, **6**, e21050.

**SUPPLEMENTARY TABLES**

**Supplementary Table 1 Behavioral tests in *Fabp3* KO mice**

**Supplementary Table 2 Behavioral tests in *Fabp5* KO mice**

**Supplementary Table 3 Behavioral tests in *Fabp7* KO mice**
